# Amyloid precursor protein controls cholesterol turnover needed for neuronal activity

**DOI:** 10.1002/emmm.201202215

**Published:** 2013-04-02

**Authors:** Nathalie Pierrot, Donatienne Tyteca, Ludovic D'auria, Ilse Dewachter, Philippe Gailly, Aurélie Hendrickx, Bernadette Tasiaux, Laetitia El Haylani, Nathalie Muls, Francisca N'Kuli, Annie Laquerrière, Jean-Baptiste Demoulin, Dominique Campion, Jean-Pierre Brion, Pierre J Courtoy, Pascal Kienlen-Campard, Jean-Noël Octave

**Affiliations:** 1 Université Catholique de LouvainBrussels, Belgium; 2 Institute of NeuroscienceBrussels, Belgium; 3 de Duve InstituteBrussels, Belgium; 4 Department of Pathology, Rouen University Hospital and ERI 28, Institute for Biomedical Research, University of RouenRouen, France; 5 Faculty of Medicine, Inserm U614-IFRMPRouen, France; 6 Department of Research, CHSRSotteville-lès-Rouen, France; 7 Laboratory of Histology and Neuropathology, Université libre de BruxellesBrussels, Belgium

**Keywords:** Alzheimer's disease, calcium oscillations, cholesterol turnover, neuron, SREBP-1

## Abstract

Perturbation of lipid metabolism favours progression of Alzheimer disease, in which processing of Amyloid Precursor Protein (APP) has important implications. APP cleavage is tightly regulated by cholesterol and APP fragments regulate lipid homeostasis. Here, we investigated whether up or down regulation of full-length APP expression affected neuronal lipid metabolism. Expression of APP decreased HMG-CoA reductase (HMGCR)-mediated cholesterol biosynthesis and SREBP mRNA levels, while its down regulation had opposite effects. APP and SREBP1 co-immunoprecipitated and co-localized in the Golgi. This interaction prevented Site-2 protease-mediated processing of SREBP1, leading to inhibition of transcription of its target genes. A GXXXG motif in APP sequence was critical for regulation of HMGCR expression. In astrocytes, APP and SREBP1 did not interact nor did APP affect cholesterol biosynthesis. Neuronal expression of APP decreased both HMGCR and cholesterol 24-hydroxylase mRNA levels and consequently cholesterol turnover, leading to inhibition of neuronal activity, which was rescued by geranylgeraniol, generated in the mevalonate pathway, in both APP expressing and mevastatin treated neurons. We conclude that APP controls cholesterol turnover needed for neuronal activity.

## INTRODUCTION

Processing of amyloid precursor protein (APP) by β- and γ-secretase activities produces β-amyloid (Aβ) peptide, which accumulates in extracellular senile plaques present in brain of patients with Alzheimer disease (AD). Although APP processing by secretase activities has been extensively studied (Esler & Wolfe, 2001), the function of the protein remains unclear. APP knockout mice have a normal phenotype with only subtle defects, resulting from functional compensation by APP-like proteins 1 and 2 (Heber et al, 2000). The γ-secretase-mediated cleavage of APP releases the APP intracellular domain (AICD), which controls the transcription of several genes [for review see (Muller et al, 2008)] by mechanisms that could involve epigenetic modifications (Huysseune et al, 2009).

Recently, genome wide association studies (GWAS) on AD confirmed that ApoE4 is a major risk factor and provided evidence for other risk genes including *Clusterin* (*CLU*) and *ABCA7* (Harold et al, 2009; Hollingworth et al, 2011; Lambert et al, 2009). The main lipoproteins in brain are ApoE and clusterin, and ABCA7 is involved in lipids efflux from cells to lipoproteins. The identification of these susceptibility loci further supports the hypothesis that perturbation of lipids metabolism (Jones et al, 2010) favours progression of AD (Shepardson et al, 2011a, b).

Cholesterol has been shown to influence APP processing and Aβ generation by modulating β- and γ-secretase activities (Fassbender et al, 2001; Grimm et al, 2008; Refolo et al, 2001; Runz et al, 2002; Yao & Papadopoulos, 2002). In turn, APP cleavage products regulate cholesterol homeostasis (Grimm et al, 2005, 2007, 2012), but the role played by full-length APP on neuronal cholesterol homeostasis remains to be investigated.

We have here studied the influence of APP expression, or down regulation of endogenous APP, on neuronal cholesterol synthesis. Cholesterol homeostasis is controlled by a family of transcription factors, known as sterol regulatory element binding proteins (SREBPs; Bengoechea-Alonso & Ericsson, 2007; Brown & Goldstein, 1999). In cells with sufficient cholesterol supply, SREBPs are transmembrane proteins retained in the endoplasmic reticulum (ER), associated with SREBP-cleavage-activating protein (SCAP), a cholesterol sensor (Feramisco et al, 2005). Upon cellular cholesterol depletion, SREBP leaves the ER to reach the Golgi, where cleavage by site-1 protease (S1P) releases the amino-terminal half of SREBP, which can be further cleaved within its membrane-spanning helix by site-2 metalloproteinase (S2P; Brown & Goldstein, 1999). The mature processed form of SREBP is released in the cytosol and can translocate into the nucleus where it modulates the expression of several genes controlling cholesterol and fatty acid homeostasis (Horton et al, 2002), including hydroxymethyl glutaryl-CoA reductase (HMGCR), HMG-CoA synthase (HMGCS), low density lipoprotein receptor (LDLR) and SREBP1/2 itself. Our results show that APP controls SREBP-mediated cholesterol biosynthesis in cultured neurons, but not in astrocytes. In neurons, down regulation of endogenous APP expression increased both cholesterol biosynthesis and hydroxylation, which were decreased following expression of APP that inhibited neuronal cholesterol turnover. Inhibition of cholesterol turnover, by either APP or mevastatin, inhibited neuronal activity measured by spontaneous and synchronous calcium oscillations (Santos et al, 2009). Apamin, a specific antagonist of the calcium-dependent potassium SK channels, and geranylgeraniol, an end product of the mevalonate pathway, rescued neuronal activity in both APP expressing and mevastatin treated neurons. These results reveal a key role of APP in the control of neuronal cholesterol turnover needed for neuronal activity. Our results illustrate an essential physiological function of APP in neurons and further emphasize the stimulation of neuronal cholesterol turnover as a possible target for the treatment of AD.

## RESULTS

### APP controls neuronal cholesterol synthesis via the SREBP pathway

**Figure 1:**
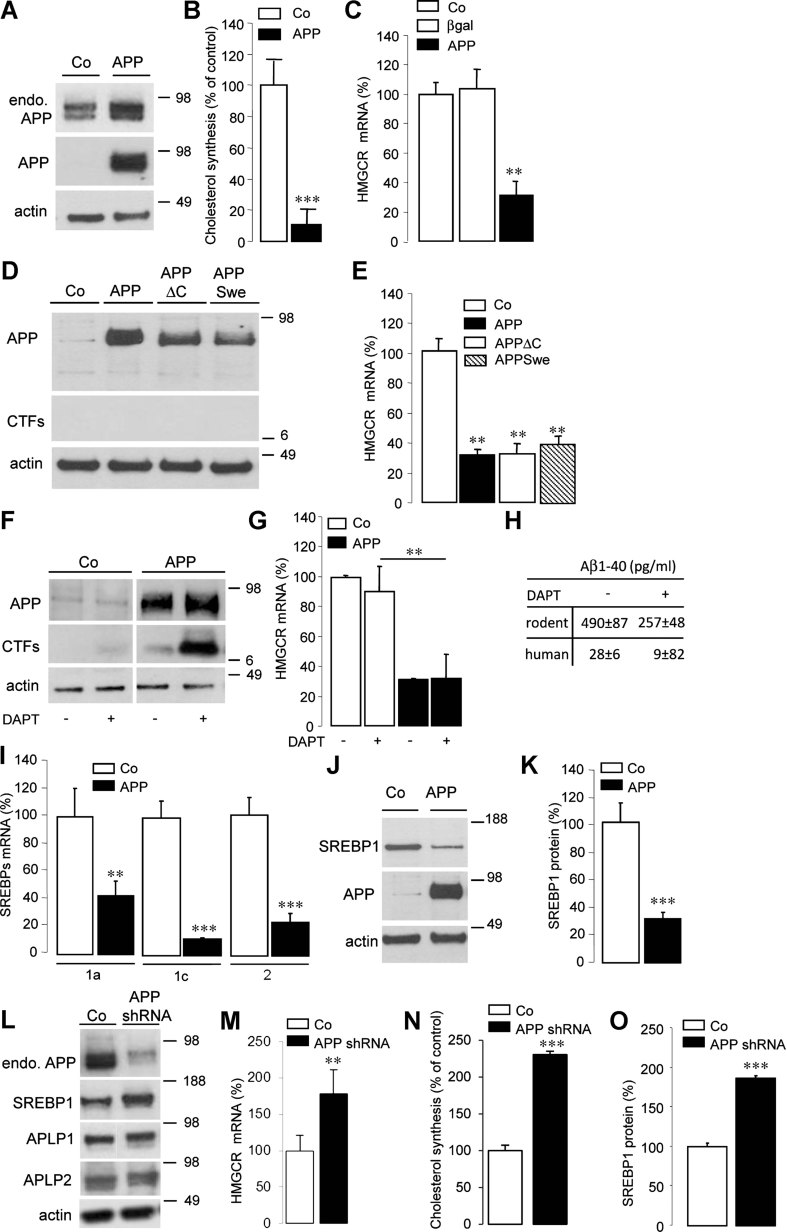
APP controls neuronal cholesterol synthesis via the SREBP pathway. Source data is available for this figure in the Supporting Information. A–O. Western blots of cell lysates from primary cultures of rat cortical neurons. Neurons were infected or not (A,D,F,J, control, Co) by AdAPP deleted or not from its C-terminal domain (APP or APPΔC, respectively, D), by AdAPPSwe carrying the Swedish mutation (APPSwe, D) and by APPshRNA1 recombinant lentivirus (APPshRNA, L). Expression of endogenous APP (endo. APP) and APP were monitored with anti-APP C-terminal and WO2 antibodies respectively (A). Cells were treated (+) or not (−) with 250 nM DAPT for 8 h and APP C-terminal fragments (CTFs) were analysed with the APP C-terminal antibody (F). Blots were further probed for the N-terminus of the SREBP1, APLP1/2 and actin (L). Cholesterol synthesis was measured by ^14^C acetate incorporation in neurons expressing APP (B) or APPshRNA (N) compared to Co (*n* = 4). Comparative qRT-PCR analyses of (C,E,G,M) HMGCR (*n* = 6) and (I) SREBP2 (*n* = 4), SREBP1a (*n* = 4) or SREBP1c (*n* = 4) mRNA levels in Co (C, E, G, I, M), APP (C, E, G, I, M), APPΔC (E), APPSwe (E), APPshRNA (M), β-galactosidase (βgal, C), APPΔC expressing neurons treated (+) or not (−) with 250 nM DAPT for 8 h (G, *n* = 6). Results were normalized by GAPDH mRNA and expressed as percentage of Co. (H) Effect of DAPT treatment on rodent and human extracellular Aβ40 release (*p* < 0.05, *n* = 6). Quantification of SREBP1/actin ratios; results were expressed as percentage of Co neurons (K, *n* = 12; O, *n* = 4), (**p < 0.01; ***p < 0.001).

As shown in Fig 1A, adenoviral expression of APP in primary cultures of rat cortical neurons increased by 60% the total APP content (1.6 ± 0.3, *n* = 5). This resulted in a major inhibition in cholesterol synthesis, measured by incorporation of ^14^C acetate (Fig 1B), readily explained by a strong reduction in HMGCR mRNA levels (Fig 1C). A significant decrease in fatty acids synthesis was also measured (Supporting Information Fig 1). The decrease in HMGCR mRNA levels was specific, not observed when neurons were infected by a control adenovirus encoding β-galactosidase (Fig 1C), occurred as soon as APP was expressed, and did not result from transient cholesterol overload, as measured by unchanged cholesterol content over time (Supporting Information Fig 2). Expression of APP carrying the Swedish mutation (APP Swe, Fig 1D), which produces more extracellular Aβ (Johnston et al, 1994), decreased HMG-CoA reductase (HMGCR) mRNA levels to similar extent as APP (Fig 1E).

Since the intracellular domain of APP (AICD) is a transcriptional regulator, we next studied whether it could account for APP-dependent repression of *HMGCR* gene transcription. Neuronal expression of APP mutants in which AICD was deleted (APPΔC; Fig 1D) decreased HMGCR mRNA levels to similar extent as full-length APP (Fig 1E).

Furthermore, inhibition of AICD release by the γ-secretase inhibitor, DAPT (Dovey et al, 2001), which induced accumulation of APP C-terminal fragments in neurons expressing full-length APP (Fig 1F) and significantly decreased both rodent and human Aβ secretion (Fig 1H), did not affect APP-mediated down regulation of HMGCR mRNA levels (Fig 1G). These two lines of evidence ruled out that APP-mediated decrease in HMGCR mRNA levels is γ-secretase or AICD dependent.

Membrane-bound transcription factors known as SREBP controls *HMGCR* gene transcription. We therefore studied whether APP expression could affect expression of different SREBP members. SREBP1a and 2 are both actors in the regulation of cholesterol synthesis. Their mRNA levels were both decreased upon neuronal expression of APP (Fig 1I). SREBP1c, which primarily controls lipogenesis, was also specifically down-regulated upon neuronal expression of APP (Fig 1I). Despite testing several anti-SREBP2 antibodies, we were unable to detect SREBP2 protein in neurons in primary cultures. However, there is important overlap between SREBP1 and SREBP2-mediated control of transcription, and high neuronal expression of SREBP1 has been demonstrated in rodent and primate brain (Ong et al, 2000). Anti-SREBP1 antibodies do not discriminate between SREBP1a and 1c produced by alternative splicing of the same gene, but revealed changes in SREBP1 protein in cellular extracts. When APP was expressed in primary cultures of rat cortical neurons (Fig 1J), a 70% significant decrease in SREBP1 was measured in cellular extracts (Fig 1K). To further investigate the ability of APP to control expression of SREBP in neurons, primary cultures of neurons were infected with lentiviruses encoding APP specific shRNA. Acute down regulation of endogenous APP expression did not modify expression levels of APLP1 and APLP2 (Fig 1L), but induced a significant increase in HMGCR mRNA levels (Fig 1M), cholesterol biosynthesis (Fig 1N) and SREBP1 expression (Fig 1O). Up regulation of SREBP1 expression in neurons from *APP* knockout (*APP*^*−/−*^) mice was much less important than that observed following down regulation of endogenous APP by shRNA. (Supporting Information Fig 3A and B). In APP knockout neurons, a very significant up regulation of APLP1 (150 ± 6%, *n* = 3) but not APLP2 was observed (Supporting Information Fig 3C), suggesting partial compensation of absent APP by APLP1. Rescue of APP by adenoviral expression of APP in *APP*^*−/−*^ neurons decreased SREBP1 by 80% (Supporting Information Fig 3A and B).

From these results, we conclude that APP controls neuronal cholesterol synthesis, via the SREBP pathway, by a γ-secretase- and AICD-independent mechanism.

### Interaction between neuronal APP and SREBP1 in the Golgi prevents S2P-mediated release of mature SREBP1

**Figure 2:**
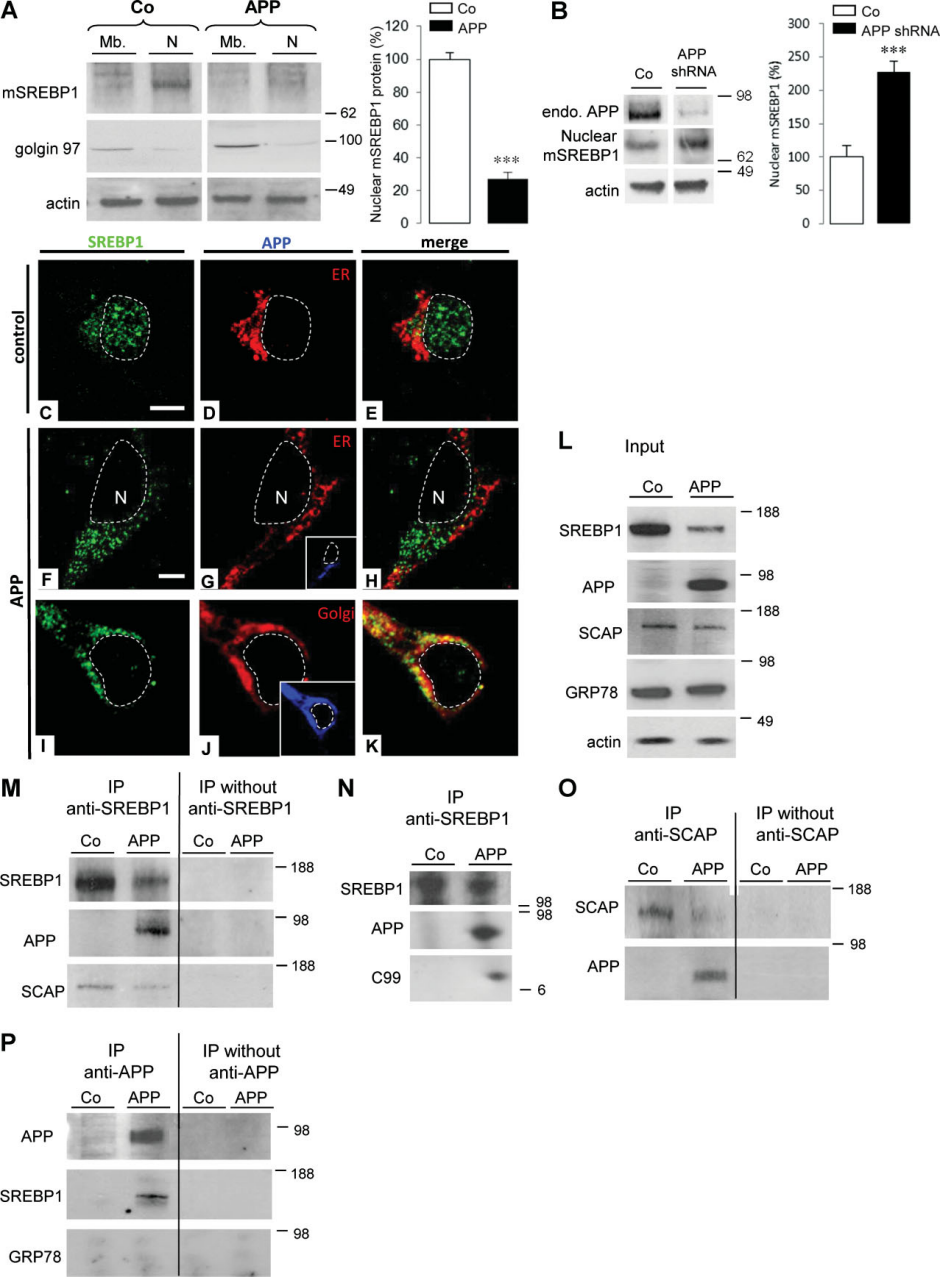
Neuronal APP and SREBP1 interaction in the Golgi prevents the release of mature SREBP1. Source data is available for this figure in the Supporting Information. A,B. Membranes (Mb) and nuclear (N) fractions from primary cultures of rat cortical neurons infected or not (control, Co) by AdAPP (APP, A) or APPshRNA1 recombinant lentivirus (APPshRNA, B) was analysed by Western blotting for the N-terminus of SREBP1 to detect mature SREBP1 (mSREBP1), stripped and reprobed for golgin 97 (Golgi), anti-APP C-terminal to detect endogenous APP (endo. APP) and actin. APP causes depletion of mSREBP1 in the nuclear fraction. C–K. Subcellular localization of SREBP1 in control (C–E) and APP-expressing neurons (F–K) were analysed by confocal microscopy with anti-N-terminus SREBP1 antibody (green). Inserts show APP expression (blue) detected with the specific WO-2 antibody (G,J). The endoplasmic reticulum (ER, red) is detected with an antibody raised against membrane-associated KDEL-bearing proteins (D,E,G,H). In APP-positive neurons, the Golgi complex (red) was labelled with an anti-TGN46 antibody (J,K), a trans-Golgi marker. (E,H,K), Merged images. Scale bar: 5 µm. L. Cell lysates from rat control neurons (Co) or expressing APP (APP) were analysed by Western blotting (input) using anti-N-terminus SREBP1, specific anti-APP, anti-SCAP, a cargo protein of SREBP1, anti-GRP78, an endoplasmic reticulum chaperon protein, and with anti-actin antibodies. M–P. Cell lysates were immunoprecipitated (IP) with or without the anti-N-terminus SREBP1 (M,N), with the anti-SCAP (O) or the specific anti-APP (P) antibodies and further analysed in Western blotting using anti-APP, -SREBP1, -SCAP or -GRP78 antibodies.

Following translocation of membrane-bound SREBP1 from the ER to the Golgi, SREBP1 is cleaved in two steps to release its soluble mature active form, mSREBP1. Nuclear translocation of mSREBP1 then activates gene transcription. Target genes include the *HMGCR/S*, *LDLR* and *SREBF1* gene itself. Since adenoviral expression of APP in neurons also repressed transcription of genes encoding LDLR and HMGCS (see Supporting information Fig 4A and B), we hypothesized that increased expression of APP in neurons would inhibit proteolytic release of mSREBP1 and/or its nuclear translocation. Following subcellular fractionation experiments, a 73 ± 4% decrease in nuclear mSREBP1 was measured in APP expressing neurons (Fig 2A). On the contrary, shRNA-mediated down regulation of endogenous APP expression induced a 127 ± 16% increase in nuclear mSREBP1 (Fig 2B).

Immunofluorescence using the H160 antibody raised against the N-terminus of SREBP1, which recognizes mSREBP1, clearly showed a nuclear localization of mSREBP1 in control neurons (Fig 2C and E). In contrast, a more cytoplasmic distribution of SREBP1 was observed in APP-expressing neurons (Fig 2F and H). These results confirmed that expression of APP in neurons not only decreased SREBP1 expression but also prevented nuclear mSREBP1 localization.

Processing into mSREBP1 requires SREBP1 translocation from the ER to the Golgi. To test whether inhibition of mSREBP1 production by APP could result from its retention together with APP in the ER, the neuronal localization of SREBP1 and APP was analysed by immunofluorescence. In APP-expressing neurons, the N-terminus of SREBP1 co-localized with APP, as well as with TGN46, a Golgi marker (Fig 2J–K). An antibody raised against the C-terminus of SREBP1 (C20) showed similar co-localization (Supporting Information Fig 5A–C), indicating that full length SREBP1 co-localized with APP in the Golgi. These results demonstrate that APP expression does not prevent SREBP1 translocation from the ER to the Golgi, but presumably causes its retention as full-length protein in the Golgi. We therefore tested for an interaction between these two proteins. APP and APP C99 were found to co-immunoprecipitate with SREBP1 (Fig 2L–N). SCAP, a SREBP-associated cargo protein, was also found in immunoprecipitates from both control and APP expressing neurons (Fig 2M). In addition, APP also co-immunoprecipitated with SCAP (Fig 2O). Moreover, SREBP1 co-immunoprecipitated with APP (Fig 2P), whereas the ER chaperone protein GRP78 did not (Fig 2N). These results indicate that SREBP1 interacts with APP and APP C99 in neurons. This interaction does not result from overexpression of APP, since the same interaction between endogenous neuronal APP and CTFs, and SREBP1 was demonstrated in co-immunoprecipitation experiments (Supporting Information Fig 6).

Altogether, these results indicated that, in neurons, interaction between APP and SREBP1 in the Golgi prevents Site-2 protease-mediated release and nuclear translocation of mature SREBP1.

### A GXXXG motif of the transmembrane domain of APP is critical for SREBP1-mediated regulation of HMGCR expression

**Figure 3:**
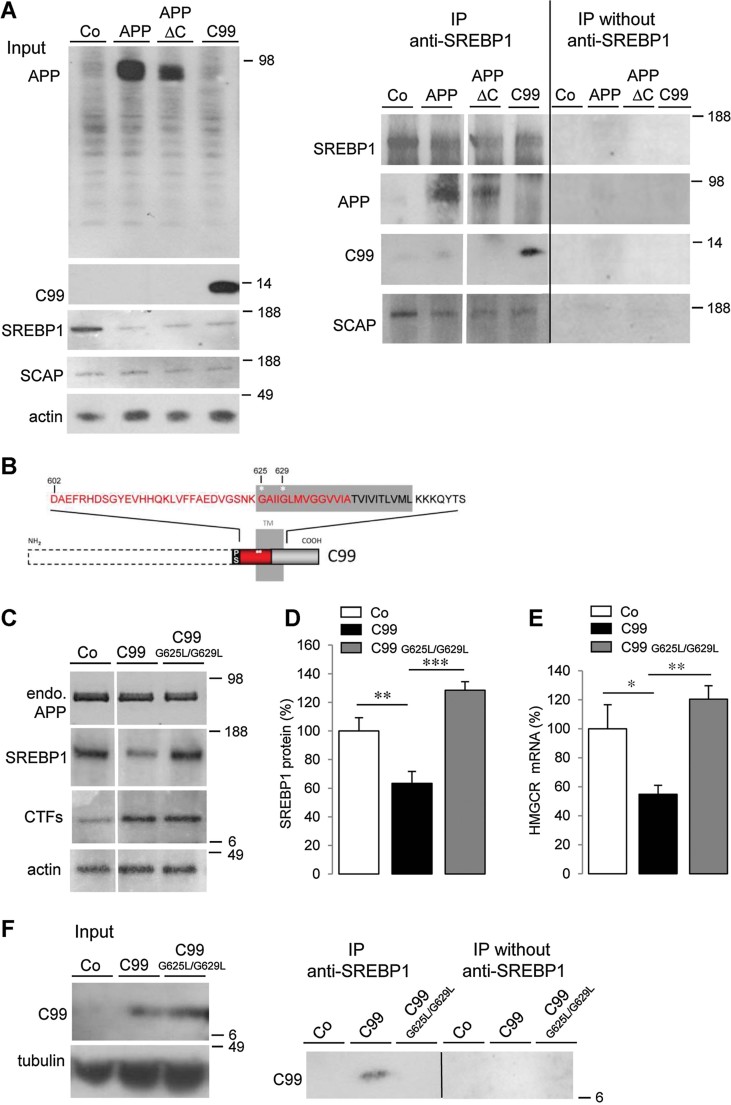
Interaction occurs between the juxta-/transmembrane domain of APP and SREBP1. Source data is available for this figure in the Supporting Information. A. Cell lysates from control neurons (Co) and neurons expressing APP or APP deleted from its C- or N- terminal domain (APPΔC or C99, respectively) were analysed in Western blotting using anti-N-terminus SREBP1, anti-APP, anti-SCAP, and anti-actin antibodies (input). Cell lysates were immunoprecipitated (IP) with or without the anti-N-terminus SREBP1 antibody and further analysed in Western blotting using anti-APP, -SCAP or -SREBP1 antibodies. B. Schematic representation of the transmembrane (TM) and juxtamembrane domains of C99 (APP 695 numbering). For the C99 mutant (G625L/G629L C99 mutant, APP 695 numbering) the two positions (625 and 629) of the amino acid substitution (Gly (G) to Leu (L)), in the first G*XXX*G motif appear in white stars. Red boxes correspond to the Aβ sequence and SP, signal peptide. C. Western blots of cell lysates from primary cultures of rat cortical neurons expressing or not (control, Co) C99 mutated or not (C99 or C99 G625L/G629L). Expression of endogenous APP (endo. APP) and C-terminal fragments (CTFs) were monitored with anti-APP C-terminal. Blots were further probed for N-terminus SREBP1 and actin. D. Quantification of SREBP1/actin ratios. Results were expressed as percentage of Co neurons (*n* = 4), (**p < 0.01; ***p < 0.001). E. Comparative qRT-PCR analyses of HMGCR (*n* = 3) mRNA levels in Co, C99 and C99 G625L/G629L expressing neurons. Results were normalized by GAPDH mRNA and expressed as percentage of Co (*p < 0.1; **p < 0.01). F. Cell lysates from neurons expressing or not (control, Co) C99 mutated or not (C99 or C99 G625L/G629L) were analysed by Western blotting (input, left panel) using WO_2_ for the detection of C99 and anti-tubulin antibodies. Cell lysates were immunoprecipitated (IP) with or without the anti-N-terminus SREBP1 and further analyzed in Western blotting using WO_2_ antibody (right panel).

In order to define the domain of APP interacting with SREBP1, rat cortical neurons were infected with recombinant adenoviruses encoding full-length APP or a mutant APP in which the C- or N-terminal domain was deleted (APPΔC or C99, respectively). In co-immunoprecipitation experiments, APP, APPΔC, and C99 were all found to interact with SREBP1 (Fig 3A). As the sequence common to these constructs is the juxta-/transmembrane domain of APP, this APP sequence seems important for APP:SREBP1 interaction, which prevents SREBP1 processing by S2P.

A recent study identified critical amino acids in the N-terminal portion of the C99 for direct binding to cholesterol (Barrett et al, 2012). In this study, structural analyses demonstrated that G700 and G704 (APP770 numbering) located in the tandem GXXXG motifs of the transmembrane domain of C99 are essential to cholesterol binding. To investigate the importance of this GXXXG motif in the APP-mediated regulation of HMGCR expression, C99 and G625L/G629L C99 mutant (APP 695 numbering; Kienlen-Campard et al, 2008; Fig 3B) were expressed in rat cortical neurons at very similar levels (Fig 3C). Contrary to the C99 mutant, neuronal expression of C99 down regulated SREBP1 levels to 63 ± 8% (*n* = 4; Fig 3C and D) and HMGCR mRNA levels to 45 ± 6% (*n* = 3; Fig 3E). Interestingly, C99, but not C99 mutant, co-immunoprecipitated with SREBP1 (Fig 3F). These results indicate that a GXXXG motif of C99, predicted from structural analysis to be essential to cholesterol binding, is critical for SREBP1-mediated regulation of HMGCR expression.

### APP does not affect S2P-mediated processing of ATF6

**Figure 4:**
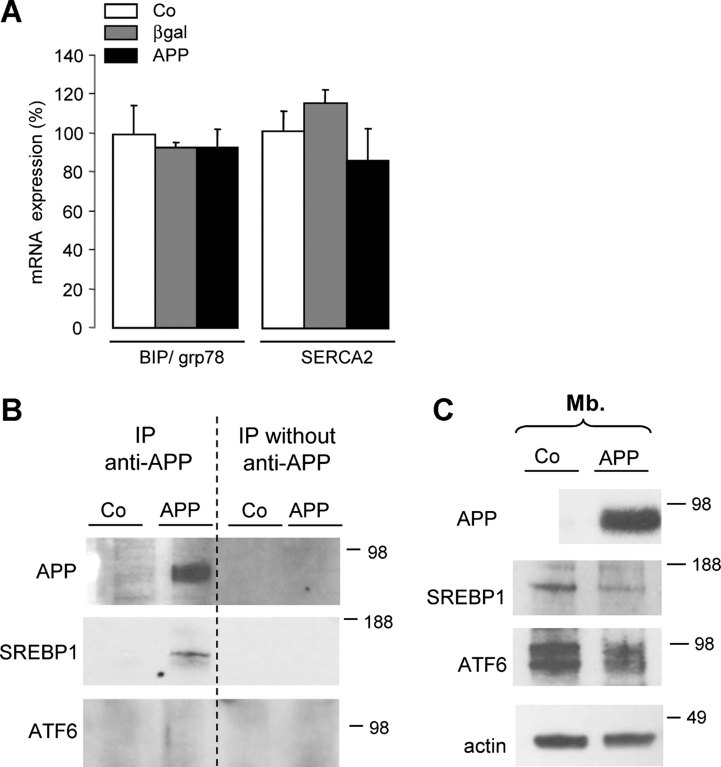
APP inhibits the cleavage of SREBP1 without affecting S2P proteolytic activity. Source data is available for this figure in the Supporting Information. A. Comparative qRT-PCR (*n* = 3) analysis of BIP/grp78 and SERCA2 mRNA in control (Co), APP and βgal expressing neurons. Results (mean ± SE) are normalized by GAPDH mRNA and expressed as percentage of Co. B. Cellular extracts from control (Co) and APP expressing neurons were immunoprecipitated with the WO-2 antibody. The presence of APP, ATF6 and SREBP1 in the immunoprecipitate was tested by Western blotting with WO-2, MBTPS2 and H160 antibodies, respectively. C. ATF6 expression in membrane (Mb.) protein fractions from control (Co) and APP expressing neurons was analysed by Western blotting with anti-ATF6 antibody. glycosylated (upper band) and non-glycosylated (lower band) forms of ATF6 were detected in both membrane fractions of Co and APP expressing neurons.

To investigate whether expression of APP would modify processing of other substrates of S2P, we measured transcription of ATF6 target genes. During ER stress, ATF6, another substrate of S2P, migrates from the ER to the Golgi (Haze et al, 1999), to be cleaved into its transcriptionally active mature form, mATF6, which also translocates into the nucleus and activates transcription of genes encoding ER-localized molecular chaperones like BIP/grp78 (Kohno et al, 1993) or the sarcoplasmic/ER Ca^2+^-ATPase 2 (SERCA2; Thuerauf et al, 2001). BIP/grp78 and SERCA2 mRNA levels were not affected by neuronal expression of APP (Fig 4A). In co-immunoprecipitation experiments, APP and ATF6 did not interact, whereas APP was able to interact with SREBP1 (Fig 4B) and glycosylated and non-glycosylated forms of ATF6 were present in membrane fractions of control and APP expressing neurons (Fig 4C), even if reduction of ATF6 was observed in APP expressing neurons. We can therefore conclude that APP expression does not induce ER stress, and does not affect S2P-mediated processing of ATF6.

### APP does not affect SREBP1-mediated cholesterol synthesis in astrocytes

**Figure 5:**
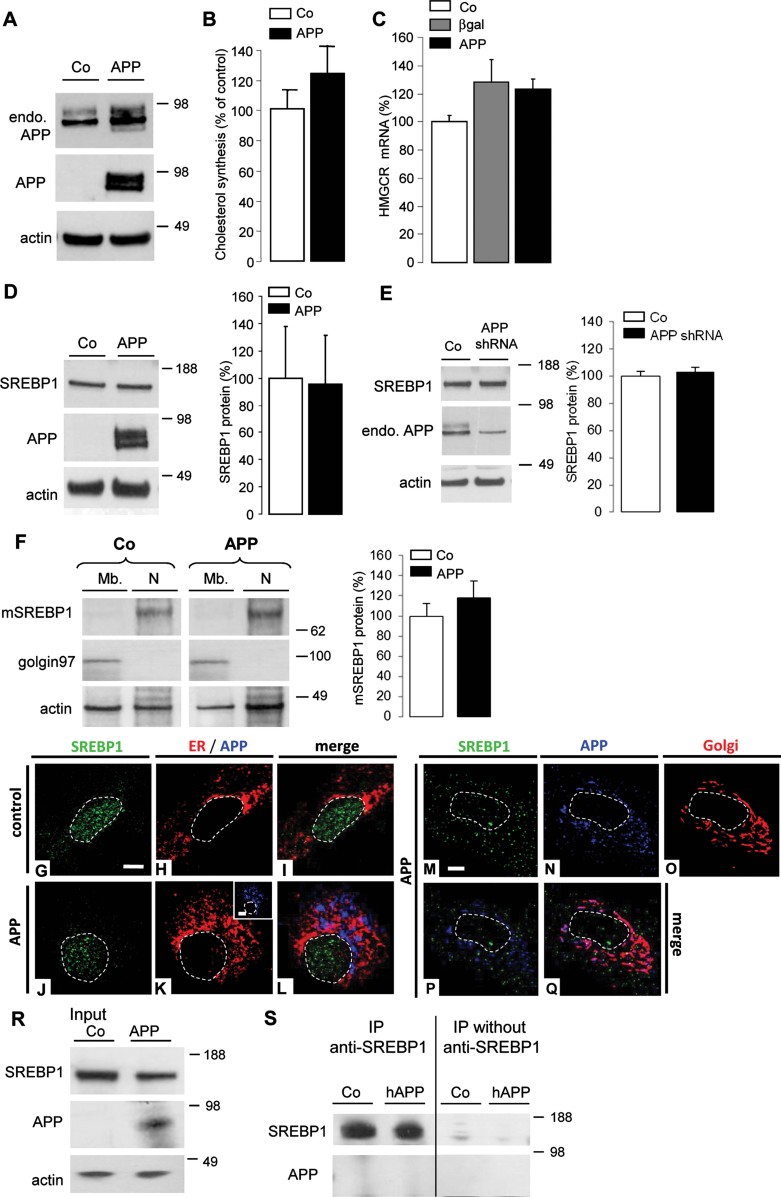
APP does not affect SREBP-mediated cholesterol synthesis in astrocytes. Source data is available for this figure in the Supporting Information. A–E. Western blots of cell lysates from primary cultures of rat cortical astrocytes infected or not (control, Co) by AdAPP (A,D, APP) or APPshRNA1 recombinant lentivirus (E, APPshRNA) were probed with antibodies to APP C-terminus (A and E), specific anti-APP (A and D), SREBP1 N-terminus (D and E) and actin (A, D and E). (B) Cholesterol synthesis measured by ^14^C acetate incorporation in APP expressing astrocytes compared to Co (*n* = 4). (C) Comparative qRT-PCR analyses of HMGCR (*n* = 3) mRNA levels in Co, APP and β-galactosidase (βgal) expressing astrocytes. Results were normalized by GAPDH mRNA and expressed as percentage of Co. (D,E) SREBP1/actin ratios were quantified and expressed as percentage of Co (*n* = 5) (right panels). F. Membrane (Mb.) and nuclear (N) fractions of Co and APP expressing astrocytes were analysed by Western blotting using an antibody raised against the N-terminus of SREBP1 to detect mature SREBP1 (mSREBP1). Blots were then probed with anti-golgin 97 and anti-actin antibodies. G–Q. Cellular localization of SREBP1 in control (G–I) and APP-expressing astrocytes (J–Q) was analysed by confocal multiplex fluorescence with anti-N-terminus SREBP1 antibody (green). Inserts show APP expression (blue) detected with the WO-2 antibody (K). The endoplasmic reticulum (ER, red) is marked for KDEL-bearing proteins (H,I,K,L). (M–Q) In APP expressing astrocytes (blue), cytoplasmic SREBP1 was labelled with an anti-Cterminus C20 (green, M,P) and Golgi apparatus with GM130 (red, O,Q) antibodies. (I,L,P,Q) Merged images. Scale bar: 5 µm. R. Cell lysates from Co and APP expressing astrocytes were analysed by Western blotting using anti-Nterminus SREBP1, -APP and -actin antibodies. S. Cell lysates were immunoprecipitated (IP) with or without the anti-N-terminus SREBP1 antibody and further analysed in Western blotting using anti-APP and -SREBP1 antibodies.

Since astrocytes are a major lipid source in the brain, we asked whether APP could also control cholesterol biosynthesis in astrocytes. In marked contrast with neurons, although adenoviral expression of APP in primary cultures of rat cortical astrocytes (Fig 5A) increased the total APP content to very similar extent as in neurons (1.5 ± 0.2, *n* = 3), it did not affect cholesterol biosynthesis, by ^14^C acetate incorporation, nor HMGCR mRNA levels (Fig 5B and C).

Expression of APP in astrocytes (Fig 5D), or down regulation of endogenous APP expression (Fig 5E), did not modify total SREBP1 content (Fig 5D and E). Similarly, expression of APP did not modify mSREBP1 in the nuclear fraction (Fig 5F), nor induce a redistribution of SREBP1, nor inhibited nuclear mSREBP1 production (Fig 5J–L). In addition, no co-localization of APP and SREBP1 could be observed (Fig 5M–Q), and these proteins did not co-immunoprecipitate (Fig 5R and S). We conclude that APP does not affect cholesterol synthesis in astrocytes, because SREBP1 and APP do not interact in these cells. This experiment further indicated that SREBP1:APP interaction observed in neurons was specific and occurred in living cells rather than after lysis.

### APP controls *in vivo* expression of SREBP1 in mice and man

**Figure 6:**
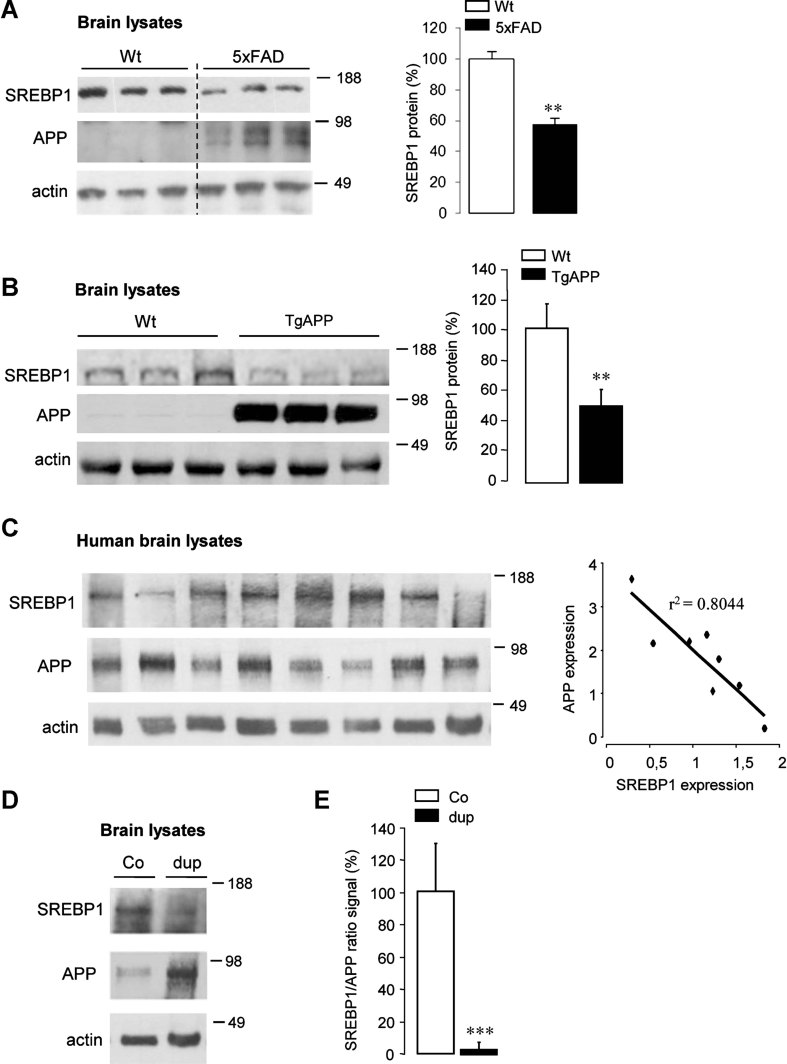
APP inversely correlates with SREBP1 in mice and man. Source data is available for this figure in the Supporting Information. A,B. Western blots analysis of brain tissue from wild type (Wt), 5×FAD mice at 15 months of age (A) and APP transgenic mice (TgAPP) at 5 months of age (B). The expression of APP was detected with anti-APP specific antibody, SREBP1 was detected with the anti-N-terminus SREBP1 antibody and protein loading was controlled using anti-actin antibody (left panels). SREBP1/actin ratios were quantified (*n* = 6, A and *n* = 3, B) and expressed as percentage of Wt controls (right panels). C. Western blot analysis of postmortem human brain homogenates with anti-APP specific antibody, SREBP1 and actin antibodies (left panel). Quantification of densitometry arbitrary units indicating an inverse correlation between APP and SREBP1 levels (right panel). D. Western blot analysis of postmortem human brain homogenates from control brain (Co) and an Alzheimer patient carrying a microduplication of the *APP* locus (dup) (left panel). The SREBP1/APP ratios were quantified on several Western blots from Co and dup brains and expressed as percentage of Co (*n* = 4) (right panel); (**p < 0.01, ***p < 0.0001).

In brain samples of 5XFAD transgenic mice, a twofold decrease in SREBP1 was found in Western blotting as compared to wild type mice from the same genetic background (Fig 6A). These mice express human APP with three different mutations (Swedish, Florida and London) as well as human PS1 with 2 different mutations (PSEN1*M146L*L286V) and develop a severe amyloid pathology at 3 months (Oakley et al, 2006). A similar decrease in SREBP1 was observed in another transgenic mouse model expressing human APP carrying the London mutation as the single transgene and developing amyloid pathology at a later age (Moechars et al, 1999; Fig 6B).

In human brain samples, SREBP1 precursor was detected as a 125 kDa protein, with different levels of expression (Fig 6C). When APP and SREBP1 signals were quantified, an inverse correlation was observed (Fig 6C). A similar inverse correlation was also observed in brain sample from an AD case with microduplication of the APP locus, in which increase in APP was concomitant with decrease in SREBP1 expression (Fig 6D and E). These results indicate that APP inversely correlates with SREBP1 in mice and man.

### APP affects neuronal cholesterol turnover and neuronal activity

**Figure 7:**
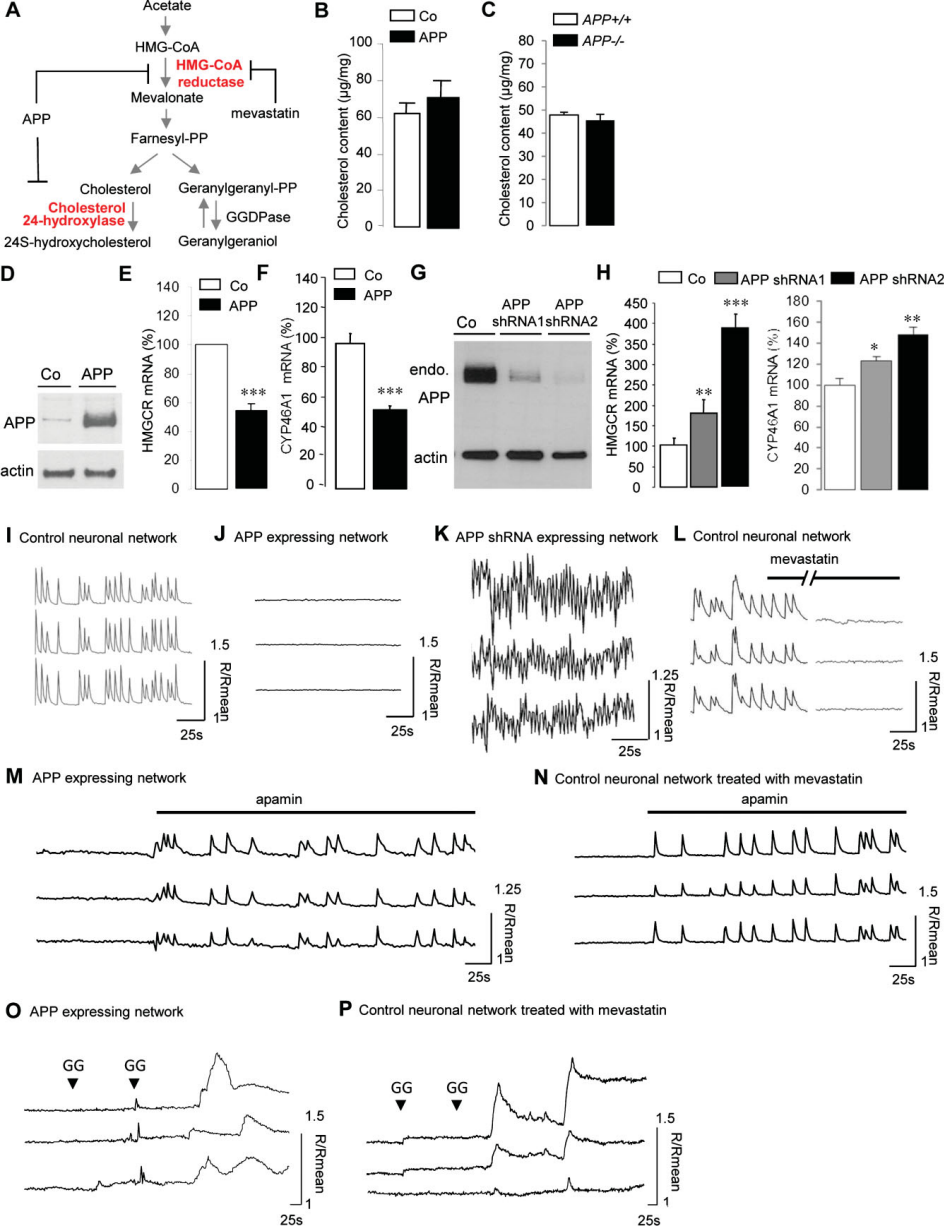
Inhibition of mevalonate pathway by APP impairs neuronal activity. Source data is available for this figure in the Supporting Information. A. Diagram of the mevalonate pathway. HMG-CoA reductase (HMGCR), the rate-limiting enzyme in the biosynthesis of cholesterol and cholesterol 24-hydroxylase, converting cholesterol into 24*S*-hydroxycholesterol, are involved in cholesterol turnover. HMGCR is inhibited by statins drugs such as mevastatin but also after APP expression. Farnesyl diphosphate (farnesyl-PP), geranylgeranyl diphosphate (geranylgeranyl-PP) and geranylgeraniol are polyisoprenoid end products of the mevalonate pathway. GGDPase (geranylgeranyl diphosphatase). A,C. Cholesterol content in cell membranes from control (Co), APP expressing neurons (APP, B) and from primary cultures of mouse cortical neurons prepared from wild type (*APP*^*+/+*^) and *APP* knockout mice (*APP*^*−/−*^, C), (*n* = 6). D–H. Western blots analysis of cell lysates from rat cortical neurons infected or not (control, Co) with AdAPP (G, APP) or APPshRNA1 and two recombinant lentiviruses (G). The expression of APP and endogenous APP (endo. APP) were detected with anti-APP specific and anti-APPC-terminal antibodies, respectively; and protein loading was controlled using anti-actin antibody. Comparative qRT-PCR analysis of HMGCR mRNA in Co, APP (E) and APPshRNA1 and 2 neurons (H, left panel) respectively. Results (*n* = 6) are normalized by GAPDH mRNA and expressed as percentage of Co. Comparative qRT-PCR analysis of CYP46A1 mRNA in Co, APP (F) and APP shRNA1 and 2 neurons (H, right panel), respectively. Results (*n* = 6) are normalized by GAPDH mRNA and expressed as percentage of Co. I–P. Traces of free cytosolic calcium concentration in three neurons of a coverslip, measured with Fura-2 AM at 13 days *in vitro* in control (I), APP (J) and APPshRNA neurons (K), or in control neurons before and 30 min (//) after addition of mevastatin (12.5 µM) (L). Simultaneous traces of [Ca^2+^] in three cells of APP expressing neurons (M), and in control neurons pretreated with mevastatin (12.5 µM, for 12 h) (N) network show that cells recover synchronous calcium oscillations after bath application of 200 nM apamin, an SK channel inhibitor. In APP expressing neurons (O), and in control neurons pretreated with mevastatin (12.5 µM) for 12 h (P), a two round of geranylgeraniol treatment (GG, 2 mM) is needed to rescue neuronal activity. Traces are expressed as *R*/*R*_mean_, were *R* is the ratio of F340/F380 and *R*_mean_ is the mean ratio value after recording.

Since expression of APP inhibits cholesterol biosynthesis specifically in neurons, we further analysed the mevalonate pathway in these cells (Fig 7A). Contrary to other cell types in the brain, neurons specifically express the *CYP46A1* gene encoding the cholesterol 24-hydroxylase (Lund et al, 1999), which transforms cholesterol into 24*S*-hydroxycholesterol. Consequently, the turnover of cholesterol is very important in neurons, resulting from equilibrium between biosynthesis and hydroxylation. Although expression of APP in neurons decreased by 90% the biosynthesis of cholesterol (Fig 7B), the content of membrane cholesterol was not affected (Fig 7B). We therefore looked at a possible down regulation of cholesterol 24-hydroxylase expression by APP. Expression of APP (Fig 7D) decreased CYP46A1 mRNA level (Fig 7F) to a very similar extent as that of HMGCR (Fig 7E). On the contrary, two different shRNA induced acute down regulation of endogenous APP expression at different levels (Fig 7G), with a concomitant and proportional up regulation of HMGCR and CYP46A1 mRNA levels (Fig 7H). In addition, genetic ablation of APP did not modify neuronal membrane cholesterol content (Fig 7C). Consequently, APP does not modify membrane cholesterol content but controls neuronal cholesterol turnover.

APP proteolytic processing not only regulates lipid metabolism (Grimm et al, 2012) but also synaptic transmission and ion channel function (Dawson et al, 1999; Wang et al, 2005). We previously reported that APP controls neuronal activity measured by spontaneous and synchronous calcium oscillations observed in mature neuronal networks (Santos et al, 2009). In this study, we observed that the resting potential of APP expressing neurons was not affected, while a significant increase in L-type calcium currents was measured in these neurons. Resulting calcium influx activated calcium-dependent potassium channels (SK channels) and increased the pic amplitude of medium after hyper polarization leading to inhibition of calcium oscillations in APP expressing neuronal networks (Santos et al, 2009). Accordingly, expression of APP in rat cortical neuronal networks inhibited calcium oscillations, while shRNA-mediated acute down regulation of endogenous APP increased the frequency of oscillations and decreased their amplitude (Fig 7I–K). To investigate whether this control of neuronal activity by APP was related to modification of cholesterol turnover, calcium oscillations were analysed in neurons treated by mevastatin, an inhibitor of HMGCR. Similar to expression of APP, a 30 min treatment with 12.5 µM mevastatin completely inhibited spontaneous calcium oscillations in 86 ± 13% of cortical neurons (214 cells analysed in five different experiments; Fig 7L). Interestingly, apamin, a specific antagonist of SK channels, was able to rescue synchronous calcium oscillations in 62 ± 6% of APP expressing neurons (117 cells analysed in three different experiments; Fig 7M) and in 65 ± 19% of neurons treated by mevastatin (112 cells analyzed in three different experiments; Fig 7N). Since the content of membrane cholesterol was not affected by APP (Fig 7B), inhibition of calcium oscillations in APP expressing neurons did not result from alteration of membrane cholesterol. Therefore, we investigated whether geranylgeraniol, the second end product of the mevalonate pathway (Fig 7A), could be involved in the generation of calcium oscillations. Indeed, a two round addition of geranylgeraniol, partially reversed inhibition of calcium oscillations induced by APP (Fig 7O) and mevastatin (Fig 7P) in 38 ± 7% of cortical neurons (195 cells analysed in four different experiments). Altogether, these results indicate that inhibition of cholesterol turnover profoundly affects neuronal activity by activating SK channels. We conclude that APP is able to control neuronal cholesterol turnover needed for neuronal activity.

## DISCUSSION

Our findings demonstrate that expression of APP at moderate levels in rat cortical neurons decreases cholesterol biosynthesis as well as mRNA levels of several genes involved in cholesterol homeostasis, namely the three SREBP isoforms *SREBP1a*, *SREBP1c*, *SREBP2*, as well as *HMGCR*, *HMGCS* and *LDLR*, which are three SREBP target genes. On the contrary, acute down regulation of endogenous APP expression in cortical neurons increases SREBP1, HMGCR mRNA levels and cholesterol biosynthesis. Among SREBP isoforms, SREBP1 and SREBP2 promote fatty acid and cholesterol synthesis (Brown & Goldstein, 1997). Since SREBP2 was undetectable in our neuronal cultures but SREBP1 has been detected in rodent brain (Ong et al, 2000), we analysed whether APP could specifically alter neuronal processing of SREBP1. We found that in neurons, interaction between APP and SREBP1 in the Golgi inhibits the S2P-mediated release of mSREBP1 and its nuclear translocation, without affecting the processing of ATF6, another substrate of S2P. In contrast, although astrocytes play a central role in the synthesis and metabolism of lipids (Herz, 2001), neither transcription of the *HMGCR* gene nor biosynthesis of cholesterol were affected by APP in primary cultures of rat cortical astrocytes. We found that APP did not affect production of mSREBP1 and its nuclear translocation because SREBP1 and APP do not interact in these cells. APP also controls SREBP1 expression *in vivo*. In transgenic mice expressing different APP mutants, down regulation of SREBP1 was observed in brain independently of APP mutations and human PS1 expression. In human brain, APP inversely correlates with SREBP1, including in an AD case with microduplication of APP locus. Recently, mRNA levels of SREBP2 were shown to be decreased in peripheral blood mononuclear cells from AD patients (Mandas et al, 2012). We also provide evidence that in neurons, APP controls cholesterol turnover by regulating both HMGCR and cholesterol 24-hydroxylase mRNA levels. Decrease in neuronal cholesterol turnover inhibits neuronal activity by activating calcium-dependent potassium SK channels.

Numerous studies have reported that modification of cholesterol content can affect APP processing. High cholesterol level favours production of Aβ (Bodovitz & Klein, 1996; Ehehalt et al, 2003; Schneider et al, 2006), by decreasing the non-amyloidogenic α-secretase activity (Bodovitz & Klein, 1996; Ehehalt et al, 2003; Schneider et al, 2006) and increasing amyloidogenic β- and γ-secretases activities (Grimm et al, 2008; Runz et al, 2002; Yao & Papadopoulos, 2002). In animal models, cholesterol also favors formation of amyloid plaques (Kuo et al, 1998; Refolo et al, 2000; Sparks et al, 1994), while cholesterol depletion reduces amyloid production and its pathological consequences (Fassbender et al, 2001; Grimm et al, 2008; Refolo et al, 2001; Simons et al, 1998). In turn, APP cleavage products, *i.e.* Aβ and AICD, have been demonstrated to modulate lipid homeostasis (Grimm et al, 2012). In particular, γ-secretase processing of APP has been reported to decrease cholesterol synthesis via inhibition of HMGCR activity and Aβ to impair SREBP2 processing (Grimm et al, 2005; Mohamed et al, 2012).

Here, we demonstrate that expression of full-length APP decreases *HMGCR* gene transcription in primary cultures of rat cortical neurons. This regulation of *HMGCR* gene transcription by APP is however independent of γ-secretase processing, as it was neither unchanged by APPΔC nor inhibited by DAPT. Inhibition of the γ-cleavage of APP prevents the production of AICD, a transcriptional regulator (Huysseune et al, 2009), which inhibits transcription of the *LRP1* gene following nuclear translocation and interaction with the *LRP1* gene promoter (Liu et al, 2007), but expression of APP without AICD (APPΔC) and inhibition of the γ-cleavage of APP both decreased transcription of the *HMGCR* gene, indicating that APP controls the transcription by another AICD-independent mechanism controlled by SREBP.

The APP juxta-/transmembrane domain is able to homo-dimerize and hetero-dimerize with other membrane proteins such as LRP1 and Notch receptors (Kinoshita et al, 2001; Oh et al, 2005; Sato et al, 2009). Not only did APP and SREBP1 co-localize in the Golgi but co-immunoprecipitation demonstrated their association through the APP juxta-/transmembrane domain, containing a GXXXG motif, which plays a key role in the control of SREBP1 and HMGCR expression. Structural analysis recently proposed that this motif in the transmembrane domain of APP is essential to cholesterol binding (Barrett et al, 2012), and in particular the second G of the GXXXG motif, which is conserved in APLPs (Aydin et al, 2012). APLP1 was able to compensate absence of APP in APP knockout neurons and APLP1 and 2 co-immunoprecipitated with SREBP1 (Supporting Information Fig 7). It was previously demonstrated that the C-terminal domain of APP, and most likely full-length APP, can form specific complexes with cholesterol at physiologically relevant cholesterol concentrations (Beel et al, 2008). Whether, in addition to SCAP, APP might function as neuronal cholesterol sensors and/or cargo proteins required for shuttling SREBP1 between ER and Golgi deserves further investigations.

Although C99 and G625L/G629L C99 mutant both contain the signal peptide of APP at their N-terminus and are therefore transported in the secretory pathway (Kienlen-Campard et al, 2008), only C99 was able to mediate SREBP1-dependent regulation of HMGCR expression, indicating the specificity of C99:SREBP1 interaction, which was further confirmed by absence of interaction between APP and ATF6, another substrate of S2P. Reduction of ATF6 expression in APP expressing neurons could be related to a direct SREBP2:ATF6 interaction allowing ATF6, by recruiting HDAC1 to the ATF6-SREBP2 complex, to antagonize SREBP2 (Zeng et al, 2004). Consequently, down regulation of ATF6 expression by APP could be considered as a retro control of expression of SREBP2 target genes.

*In vivo*, cortical neurons form oscillating networks of various sizes involved in temporal representation and long-term consolidation of information (Buzsaki & Draguhn, 2004). In neuronal networks in culture, control of neuronal cholesterol turnover by APP had important consequences on neuronal activity. In APP expressing neuronal networks, activation of SK channels was involved in the inhibition of calcium oscillations induced by alteration of cholesterol turnover. It was recently demonstrated (Campia et al, 2012) that enhancing the activity of the mevalonate pathway in cardiomyocytes increases the cellular levels of ubiquinone, and the synthesis of ATP, which can inhibit SK channels (Jiang & Haddad, 1997). A decrease in ATP, resulting from inhibition of the mevalonate pathway, could significantly activate SK channels, which have to be inhibited by apamin to rescue calcium oscillations. Interestingly, our preliminary data indicate a significant decrease in ATP level in APP expressing neurons.

In conclusion, our data identify a novel role of APP in neurons, *i.e.* control of cholesterol biosynthesis and hydroxylation and consequently cholesterol turnover that is crucial for synaptic function. Synaptic activity is regulated by small GTPases proteins (Schubert & Dotti, 2007), and the isoprenoids farnesyl-PP and geranylgeranyl-PP serve as substrates for their prenylation (Hooff et al, 2010). We provide evidence that geranylgeraniol, an end product of the mevalonate pathway, is able to rescue APP-mediated inhibition of synaptic activity. Interestingly, alteration of neuronal cholesterol turnover in CYP46A1 knockout mice induces defects in memory and LTP induction and maintenance (Kotti et al, 2006). In both CYP46A1 knockout mice and APP expressing neurons, production of 24S-hydroxycholesterol, the major neuronal agonist of LXR receptors, is inhibited. Synaptic failure in AD is well established (Selkoe, 2002), and our results argue for stimulation of cholesterol turnover to rescue APP-mediated inhibition of neuronal activity. Such stimulation could be performed by activation of LXR receptors, which could favour neuronal cholesterol synthesis and efflux (Cao et al, 2007). In addition, retinoid X receptor activation was recently demonstrated to stimulate Aβ clearance in an ApoE-dependent manner (Cramer et al, 2012). Together these data indicate the relevance of this novel identified function of APP in neurons, and underline the need to further assess its pathological significance and therapeutic potential.

## MATERIALS AND METHODS

### Animals and tissues samples

All animal procedures used in the study were carried out in accordance with institutional and European guidelines as certified by Animal Ethics Committee. 5xFAD mice (Oakley et al, 2006) were obtained from Jackson Laboratories (strain: B6SJL-Tg (APPSwFlLon, PSEN1*M146L*L286V) 6799Vas/Mmjax) and backcrossed to C57Bl6/J wild type (Wt) mice. C57Bl6/J Wt mice were used as control animals. Experiments on 5xFAD mice and age-matched control were performed at 15 months of age. One hemisphere was frozen in liquid nitrogen and stored at −80°C for biochemical analysis.

Brains from 5-month-old wild type and transgenic APP mice (TgAPP expressing hAPP 695 isoform carrying the London mutation) were excised, frozen in liquid nitrogen, and stored at −80°C until analysis. Samples of TgAPP mice (Moechars et al, 1999) were obtained from reMYND NV (spin-off University Leuven, Belgium).

Postmortem human brains (*n* = 8) were collected with the approval of the Ethical Committee at the Medical School of the Free University of Brussels. One hemisphere was frozen in liquid nitrogen and stored at −80°C for biochemical analysis. The other hemisphere was fixed by immersion in 10% formalin (v/v) for neuropathological analysis. Postmortem frozen temporal brain tissue from an autosomal dominant early-onset AD patient with APP microduplication was obtained from the Department of Pathology (Rouen University Hospital, France). Genetic analyses, clinical and neuropathological evaluations were previously reported (Cabrejo et al, 2006; Rovelet-Lecrux et al, 2006). Briefly, 1 cm coronal sections were performed on the right cerebral hemisphere at the time of autopsy, and immediately frozen at −80° until use. Neuropathological examination was performed on multiple formalin fixed, paraffin embedded brain tissue samples. This case was characterized by severe amyloid angiopathy and AD lesions corresponding to Braak stage V–VI (Braak & Braak, 1991). In this family, the microduplication corresponded to a 780 kb segment mapping to chromosome 21q2.1, including the *APP* locus with no contiguous gene.

### Reagents and antibodies

All cell culture reagents were purchased from Invitrogen (Carlsbad, CA). Antibodies were purchased as indicated: mouse monoclonal WO-2 anti-hAPP and rabbit polyclonal anti-APLP1 and APLP2 antibodies (Millipore, Billerica, MA); rabbit polyclonal anti-APP C-terminus and β-actin (Sigma–Aldrich, St-Louis, MO); anti-SREBP1 N-terminus H-160 (Santa Cruz Biotechnology, La Jolla, CA); anti-KDEL-bearing proteins, TGN46, GM130, golgin 97, SCAP K-19 and GRP78 (Abcam, Cambridge, UK).

### Cell cultures

Primary cultures of cortical neurons were prepared from 17 to 18-day-old Wistar rat embryos. Cells were plated in culture dishes (4 × 10^5^ cells/cm^2^) pre-treated with 10 µg/ml poly-L-lysine in phosphate buffered saline (PBS) and cultured for 6 days *in vitro* in neurobasal medium supplemented with 2% v/v B-27 medium and 0.5 mM L-glutamine prior to infection with recombinant adenoviruses. The cultures were maintained at 37°C in a 5% CO_2_ atmosphere. Under these conditions, neuronal cultures contain more than 90% neuronal cells, which display high differentiation and survival rate (Brewer, 1995).

Primary cultures of cortical neurons were prepared from P0–P1 newborn wild type and *APP*^*−/−*^ mice. Cortices were dissected in neurobasal medium supplemented with 2% v/v B-27 medium, 0.5 mM L-glutamine and penicillin–streptomycin (50 µg/ml of each), and immediately digested with a trypsin solution (220 u/mg) containing DNAse (1 mg/ml) at 37°C for exactly 3 min. After removal of the trypsine/DNAse solution, cortices were further dissociated in neurobasal medium supplemented with DNAse (0.5 mg/ml). Cells were plated (4 × 10^5^ cells/cm^2^) and cultured for 6 days *in vitro* prior to infection with recombinant adenoviruses (Ad).

Primary cultures of cortical astrocytes were prepared from newborn Wistar rats as previously described (Vermeiren et al, 2005). Briefly, cortices were isolated and dissociated in Dulbecco's modified Eagle medium (glutaMAX) supplemented with 10% foetal calf serum (FCS), proline (50 µg/ml), penicillin–streptomycin (50 µg/ml) and fungizone (2.5 µg/ml). After centrifugation, cells were seeded into 75 cm^2^ culture flasks and grown at 37°C in a 5% CO_2_ atmosphere. After 7 days, oligodendrocytes were eliminated by shaking. Three days later, cells were plated at 10^4^ cells/cm^2^ in culture dishes pre-treated with 10 µg/ml poly-L-lysine in PBS. After 2 days, cell differentiation was initiated by decreasing FCS concentration to 3%.

### Recombinant viruses and infection

Recombinant adenoviruses encoding β-galactosidase (Adβgal), human APP695 without its C-terminal region (AdAPPΔC) or human APP carrying the Swedish mutation (AdAPPSwe) was described previously (Lemarchand et al, 1992). The APPΔC recombinant adenovirus construct contains a stop codon after the three lysine residues that follow the transmembrane domain. Wild-type human APP695 (AdAPP) and AdC99 were constructed and purified using the AdEasy™ XL Adenoviral Vector System (Stratagene, La Jolla, CA). The C99 recombinant adenovirus construct corresponds to the β-cleaved C-terminal fragment of hAPP fused to the APP signal peptide sequence. The C99 recombinant lentivirus harbouring the G625L/G629L mutations was constructed as previously described (Kienlen-Campard et al, 2008). After 6–7 days in culture, cells were infected at a multiplicity of infection of 10 in a minimal volume of culture medium for 4 h. Then, infection medium was replaced by fresh culture medium for 4 days. In these conditions, the recombinant viruses infected about 90% of cells.

Two different plasmids encoding shRNA raised against APP mRNA (APPshRNA1 and APPshRNA2) were obtained from Sigma–Aldrich (St-Louis, MO) and used for construction of recombinant lentiviruses, as previously described (Salmon & Trono, 2007). After 7 days *in vitro*, APP expression was down-regulated in neurons by infection with lentivirus for 3 days before analysis. In experiments using shRNA lentiviruses, a recombinant lentivirus encoding the neomycin resistance gene was used as a negative control.

### Treatments

Four days after infection, cells were treated for 8 h with 250 nM DAPT, a functional γ-secretase inhibitor (a kind gift from L. Mercken, Aventis, Paris, France) or for 12 h with 12.5 µM mevastatin, a HMGCR inhibitor (compactin, Sigma–Aldrich, St-Louis, MO). A 100 µM stock solution of compactin was prepared exactly as previously described (Kita et al, 1980).

For cytosolic free Ca^2+^ measurement, acute treatment of neurons with 200 nM apamin (Sigma–Aldrich; St-Louis, MO) was performed using perfusion of the incubation chamber. Rescue of calcium oscillations by geranylgeraniol (Sigma–Aldrich, St-Louis, MO) was performed at 2 mM, respectively. Geranylgeraniol was first dissolved in a small volume of ethanol as previously described (Kotti et al, 2008).

### Aβ measurements

Human and rodent Aβ40 were determined in the same cell culture supernatants using the Multi-Spot Human and Rodent (6E10 and 4G8, respectively) Aβ Triplex Assay and theSECTOR Imager 2400 (Meso Scale Discovery) according to manufacturer's instructions.

### Cholesterol extraction and assay

Cholesterol was extracted and purified as previously described (Bligh & Dyer, 1959). Briefly, 4 days after infection, cells were harvested in water and lipids were extracted with 3 volumes of chloroform/methanol (2:1 v/v), stirred 1 min and centrifuged at 1760 *g* for 15 min. The organic phase was collected and washed with 2 volumes of 0.05 M NaCl, then twice with 2 volumes of 0.36 M CaCl_2_/methanol solution (1:1 v/v) and centrifuged at 1760 *g* for 15 min. Organic phase was collected, dried under argon, and harvested in 0.5% Triton X-100. Cholesterol was assayed using the cholesterol oxidase-base Amplex Red Cholesterol Assay kit (Molecular Probes™, Invitrogen) and cholesterol esterase was omitted from the assay to exclude intracellular cholesterol esters.

### Cholesterol synthesis

Cholesterol synthesis was measured 4 days after infection. Cells were washed with PBS and [1-^14^C] acetate (2.5 µCi in 0.5 ml) was added for 4 h. After washing, cells were scraped, collected at 150 *g* for 5 min, resuspended in PBS (200 µl) and solubilized by addition of 20 volumes of chloroform/methanol (2:1 v/v). Four volumes of 0.9% NaCl were added to separate the two phases. The chloroform phase was dried under a nitrogen flux, hydrolyzed for 1 h at 80°C in 1 ml of methanol containing 1 M KOH, then hexane was added for extraction. The upper phase was collected, dried and analysed by thin layer chromatography using silica gel 60 plate (Merck, Darmstadt, Germany), which was developed with hexane/diethyl ether/acetic acid (87:20:1 by volume), with reference to internal cholesterol standard (Sigma, St Louis, MO). Radioactivity was quantified using an Instant Imager (PerkinElmer, Waltham, MA) and standards were visualized in iodine vapours. The radioactivity was further measured in a β-counter. Results were normalized to total protein content.

### Western blotting

Cells lysate (10 µg protein) were analysed by Western blotting using 4–12% Nupage™ bis–Tris gels (Invitrogen). Nitrocellulose membranes were incubated overnight at 4°C with the following primary antibodies: human APP-specific WO-2 (1:2000); anti-APP C-terminal (1:2000); anti-N-terminus of SREBP1 H-160 (1:1000); anti-APLP1 (1:4000); anti-APLP2 (1:2000); anti-golgin97 (1:100); anti-SCAP K-19 (1:1000); anti-GRP78 (1:100); anti-β-actin (1:2000) and anti-ATF6 (1:1000). Blots were incubated with horseradish peroxidase-conjugated secondary antibodies, revealed by ECL (Amersham Pharmacia), and quantified using the Quantity One™ software (Bio-Rad Laboratories, Hercules, CA). Actin was used as internal standard to normalize protein load in gels.

### Subcellular fractionation

Cells were harvested on ice in 0.25 M sucrose containing 1 mM EDTA, 3 mM imidazole buffered at pH 7. Cell suspension was homogenized in a tight Dounce homogenizer. A low-speed nuclear fraction was pelleted at 1000 *g* for 10 min and extracted ten times by resuspension and sedimentation. A high-speed membrane fraction of pooled postnuclear supernatants was further sedimented at 100,000 *g* for 60 min in a Ti50 rotor (Beckman). Nuclear and membrane fractions were analysed by Western blotting using 4–12% Nupage™ gels as above.

### Immunoprecipitation

Neurons or astrocytes were scraped and pelleted in cold PBS. Cells were solubilized in immunoprecipitation buffer (25 mM Tris pH 6.8, 0.5% Triton X-100, 0.5% Nonidet P-40) containing a protease inhibitor cocktail (Calbiochem, San Diego, CA). Samples were immunoprecipitated (500 µg of proteins) with 2 µg H-160, WO-2 or SCAP K19 antibodies overnight at 4°C. Protein A-sepharose (50 mg/ml, Amersham Pharmacia) was then added for 4 h. The samples were centrifuged at 15,800 *g* for 2 min at 4°C, and pellets were washed twice with immunoprecipitation buffer and once with TBS buffer (10 mM Tris, pH 7.5). Pellets were incubated for 5 min at 95°C in sample buffer (125 mM Tris pH 6.8, 20% glycerol, 4% SDS, 10% β-mercaptoethanol, 1% bromophenol blue). Samples were centrifuged at 15,800 *g* for 2 min at 4°C and supernatants were analysed by Western blotting using 4–12% Nupage™ gels as above.

### Immunofluorescence

Neurons or astrocytes were seeded at 10^5^ cells/cm^2^ on glass coverslips, fixed with 4% v/v formaldehyde at room temperature then permeabilized with 1% Triton X100 (v/v) in PBS both for 15 min. Non-specific binding was prevented by 1 h preincubation in PBS/3% non-fat dry milk, followed by 1 h incubation with primary antibodies: H-160 (1:100); WO-2 (1:1000); anti-GM130 (1:50), anti-TGN46 (1:50) and anti-KDEL (1:50). After three washes with PBS, samples were incubated for 1 h with 5 µg/ml Alexa-labelled secondary antibodies (Molecular Probes, Invitrogen). After three additional washes, preparations were mounted in Moviol and examined with a LSM 510 META confocal microscope (Zeiss, Jena, Germany) using a Plan-Apochromat 63X/1.4 oil DIC objective.

### RNA extraction and real time PCR

Total RNA was isolated by TriPure Isolation Reagent according to the manufacturer's protocol. RNA samples were resuspended in DEPC-treated water. Reverse transcription was carried out with the iScript cDNA synthesis Kit, using 1 µg of total RNA in a total volume reaction of 20 µL. Controls were performed without reverse transcriptase to rule out amplification of contaminant genomic DNA. Real-time PCR was performed for the amplification of HMGCR and glyceraldehyde phosphate dehydrogenase (GAPDH) cDNAs. Primers were purchased from Sigma–Aldrich (St-Louis, MO); F, Forward primer; R, Reverse primer:

HMGCR
F-5′-TGCCTGCAGATGCTAGGTGTT-3′
R-5′-CACACAATTCGGGCAAGCT-3′
CYP46A1
F-5′-GGCTAAGAAGTATGGTCCTG-3′
R-5′-CTTGGTGGACATCAGGAACT-3′
BIP/grp78
F-5′-TGACCTGGTTCTGCTTGATG-3′
R-5′-TCTTTTGTCAGGGGTCGTTC-3′
SERCA2
F-5′-TCCATCTGCTTGTCCATGTC-3′
R-5′-ACAGGCAGGGAGATTTTCAG-3′
GAPDH
F-5′-CCCCCAATGTATCCGTTGTG-3′
R-5′-TGATTTCCCGTAGGACCGAT-3′

Primers used for the amplification of SREBP1a, SREBP1c and SREBP2 cDNAs were kindly obtained from E. Lefai, INSERM, Lyon, France (Gosmain et al, 2005).

Real-time PCR was carried out in a total volume of 25 µl containing 2 ng cDNA template, 0.3 µM of the appropriate primers and the IQ™ SYBR® Green Supermix 1×. The PCR protocol consisted of 40 amplification cycles (95°C for 30 s, 60°C for 45 s and 79°C for 15 s) and was performed using an iCycler IQ™ multicolor Real-Time PCR detection system (Bio-Rad). For quantification, a relative standard curve was determined in the same conditions for each target gene with a fourfold dilution (from 100 to 0.097 ng) of a cDNA template mix. Each sample was normalized with the relative expression of GAPDH. Calculation of *C*_t_, standard curve preparation and quantification of mRNA in the samples were performed by the ‘post run data analysis’ software provided with the iCycler system (Bio-Rad).

**The paper explained**

**PROBLEM**

Alzheimer disease (AD) is characterized by the presence in the brain of two types of lesions corresponding to intraneuronal neurofibrillary tangles, and extracellular senile plaques, containing an amyloid deposit of Aβ peptide produced from the APP. The amyloid cascade hypothesis assumes that Aβ plaques cause dementia. However, anti-Aβ therapies in humans have, so far, failed. Although processing of APP has been extensively studied, the neuronal function of the protein remains unclear. GWAS on AD recently confirmed that the epsilon 4 allele of the ApoE gene is a major risk factor and provided evidence for other risk genes encoding proteins involved in cholesterol homeostasis. These susceptibility loci further support the hypothesis that perturbation of lipids metabolism favours progression of AD. We studied whether APP is able to control cholesterol homeostasis.

**RESULTS**

The biosynthesis of cholesterol is regulated by SREBP. In cultured neurons, interaction between APP and SREBP controls production the mature SREBP transcription factor and consequently expression of its target genes, including the gene encoding HMGCR, the limiting enzyme in biosynthesis of cholesterol. Therefore, expression of APP inhibits biosynthesis of cholesterol while down regulation of endogenous APP has the opposite effect. However, APP, under conditions of excess or defect, does not affect neuronal membrane cholesterol content. This results from the APP-mediated control of the expression of cholesterol 24-hydroxylase, a neuronal specific enzyme involved in hydroxylation of cholesterol. Consequently, APP controls cholesterol turnover. Such a regulation does not occur in astrocytes, in which APP and SREBP do not interact.

The control of neuronal cholesterol turnover by APP in the mevalonate pathway has important consequences on neuronal activity. APP and mevastatin both reduce cholesterol synthesis and turnover, leading to inhibition of neuronal activity by activation of calcium-dependent potassium SK channels. Apamin, a specific antagonist of SK channels, and geranylgeraniol produced in the mevalonate pathway, are able to rescue neuronal activity in both hAPP expressing and mevastatin treated neurons.

**IMPACT**

Our study provides new insight in an important function of APP, which is able to control neuronal cholesterol turnover needed for neuronal activity. These findings have important clinical applications, as they offer therapeutical alternatives for AD treatment based on the function rather than the processing of APP. Cholesterol turnover can be stimulated by activation of nuclear receptors of the liver X receptor (LXR) family, which are activated by oxysterols, and in particular by 24-S-hydroxycholesterol produced in neurons under the control of APP. Such activation could improve the production of end products generated in the mevalonate pathway in order to prevent synaptic dysfunctions underling learning and memory defects observed in AD. Activation of RXR was recently demonstrated to induce ApoE-mediated clearance of Aβ and to reverse deficits in a mouse model of AD, further emphasizing the stimulation of neuronal cholesterol turnover as a possible target for the treatment of dementia.

### Cytosolic free Ca^2+^ measurement in single neurons

Neurons were plated at a density of 1.8 × 10^5^ cells/cm^2^ on coated 22 mm round glass coverslips. Four days after adenoviral infection, neurons were incubated in the dark in the presence of the Ca^2+^ indicator fura-2 acetoxymethylester (Fura-2 AM; Calbiochem, Camarillo, CA, USA) at a final concentration of 2 µM in Krebs-HEPES buffer (10 mM HEPES, 135 mM NaCl, 6 mM KCl, 2 mM CaCl_2_, 1.2 mM MgCl_2_, 10 mM glucose, pH 7.4) for 30 min at room temperature. Coverslips were then washed and mounted in a heated (37°C) microscope chamber (1 ml). Cells were alternately excited (1 or 2 Hz) at 340 and 380 nm for 100 ms using a Lambda DG-4 Ultra High Speed Wavelength Switcher (Sutter Instrument, Novato, CA) coupled to a Zeiss Axiovert 200 M inverted microscope (X20 fluorescence objective; Zeiss Belgium, Zaventem, BE). Images were acquired with a Zeiss Axiocam camera coupled to a 510 nm emission filter and analysed with the Axiovision software. Calcium concentration was evaluated from the ratio of fluorescence emission intensities excited at the two wavelengths. Baseline mean ratio value (*R*_mean_) was the mean ratio value after recording. Calcium oscillations expressed as *R*/*R*_mean_ were defined as variations of more than 10% from *R*_mean_, occurring synchronously in several cells of the field.

### Statistical analysis and presentation of the results

The number of samples (*n*) in each experimental condition is indicated in figure legends. An unpaired Student's *t* test is applied when the statistical units underlying two samples being compared are non-overlapping and a one-way analysis of variance (ANOVA) followed by Bonferroni's multiple-comparison post-test is applied when many subgroups are compared. Differences were considered significant at *p* < 0.05 (*p < 0.1, **p < 0.01; ***p < 0.001). Each Western blot shown is representative of at least three independent experiments. For LTP experiments and Aβ assay, statistical analysis was done with Student's *t*-test on fEPSP slope values post stimulation.

## Author contributions

NP and JNO designed research; NP performed primary cultures, infection and biochemical experiments, production of shRNA and viruses, calcium oscillations analysis; NP and DT performed immunofluorescence; LA conducted measurements of cholesterol synthesis; ID provided 5XFAD mice; PG provided inverted microscope for calcium imaging; AH provided APP knockout mice; BT and LH quantified Aβ by MSD, NM analysed the effect of shRNA on astrocytes, FK performed subcellular fractionation; AL, DC and JPB provided samples of Alzheimer's patients, JBD designed peptides. All authors critically discussed results. NP and JNO wrote the manuscript with critical evaluation by ID, PJC and PKC.

## For more information

http://www.uclouvain.be/en-ions.html

## Notes

As a service to our authors and readers, this journal provides supporting information supplied by the authors. Such materials are peer reviewed and may be re-organized for online delivery, but are not copy-edited or typeset. Technical support issues arising from supporting information (other than missing files) should be addressed to the authors.
