# Supplementary material for: Amyloid precursor protein controls cholesterol turnover needed for neuronal activity
Source: EMBO Mol Med. 2013 Apr 2;5(4):608–25. doi: 10.1002/emmm.201202215 (PMC3628100; doi:10.1002/emmm.201202215)
Supplement: Supplementary file 1 [file emmm0005-0608-sd1.pdf]

EMBO Molecular Medicine

Amyloid Precursor Protein Controls Neuronal Cholesterol Turnover Needed For Synaptic Activity

Nathalie Pierrot, Donatienne Tyteca, Ludovic D'auria, Ilse Dewachter, Philippe Gailly, Laurence Ris, Aurelie Hendrickx, Bernadette Tasiaux, Laetitia El Haylani, Nathalie Muls, Francisca N'Kuli, Annie Laquerrière, Jean-Baptiste Demoulin, Dominique Campion, Jean-Pierre Brion, Pierre J. Courtoy, Pascal Kienlen-Campard and Jean-Noël Octave

Corresponding author: Jean-Noel Octave, Universite catholique de Louvain

Review timeline:

Submission date:	29 June 2012
Editorial Decision:	08 August 2012
Resubmission:	26 October 2012
Editorial Decision:	17 December 2012
Revision received:	21 January 2013
Accepted:	06 February 2013

Transaction Report:

(Note: With the exception of the correction of typographical or spelling errors that could be a source of ambiguity, letters and reports are not edited. The original formatting of letters and referee reports may not be reflected in this compilation.)

Editor: Anneke Funk / Natascha Bushati / Céline Carret

1st Editorial Decision 08 August 2012

Thank you for the submission of your manuscript "Amyloid Precursor Protein Controls Neuronal Cholesterol Turnover Needed For Synaptic Activity" and please accept my apologies for the delayed reply. We have now finally heard back from the three referees whom we asked to evaluate your manuscript. As you will see, the referees acknowledge that the overall topic of the manuscript is potentially interesting and reviewer #2 is rather positive about the manuscript. However, reviewers #1 and #3 are much more reserved and raise significant concerns, which, I am afraid, preclude publication of the manuscript in EMBO Molecular Medicine.

As you will see, the referees appreciate the relevant topic. However, specifically reviewers #1 and #3 raise important criticisms, in terms of both the conceptual advance provided and the conclusiveness of the results. These reviewers feel that the findings are not sufficiently well supported by the data and that extensive additional experiments would be needed to address the concerns. As such, I am afraid that two out of three referees would not support publication of the manuscript. Thus, we feel that the level of support provided by the reviewers remains too limited and I see no choice but to return the manuscript with the message that we cannot offer to publish it.

Given the potential interest of the findings, we would, however, have no objection to consider a new manuscript on the same topic if at some time in the near future you obtained data that would

considerably strengthen the study and convincingly address the reviewers' concerns in full. I would like to stress, however, that if you were to send a new manuscript, this would be treated as a new submission rather than a revision and would be reviewed afresh, in particular with respect to the literature and the novelty of your findings at the time of resubmission. If you decide to follow this route, please nevertheless upload a letter of response to the reviewers' comments.

I am sorry to have to disappoint you at this stage but hope that this negative decision does not prevent you from considering EMBO Medicine for the publication of future studies and that the reviewers' comments are helpful in your continued work in this area.

***** Reviewer's comments *****

Referee #1:

Generally speaking, this is an interesting and well written manuscript with sound biochemical data. It provides convincing evidence that APP expression affects cholesterol turnover via sterol regulatory element binding proteins (SREBP) in neurons. Basically, all the experiments have been done in two different ways (overexpression of human APP and APP knockout) to prove the role of APP in cholesterol biosynthesis. The biochemical results are very convincing as the co-localization of APP and SREBP1 in the Golgi and the identification of a possible interaction domain of APP with SERBP1. However, the important link between the biochemical observations and synaptic activity is not convincingly supported by the data presented: The demonstration that geranylgeraniol, an end product of the mevalonate pathway, rescues defects in calcium oscillations in cultured rat cortical neurons expressing human APP, as well as LTP defects in 5xFAD transgenic mice is by no means sufficient to convince this reviewer that APP and its proposed function in the cholesterol turnover is indeed crucial for synaptic function. An inhibition of the mevalonate pathway by APP may for example simply affect the resting membrane potential of neurons. A detailed electrophysiological analysis is needed to support the conclusions drawn by the authors.

Referee #2 (Comments on Novelty/Model System):

The bulk of the manuscript uses cultured neurons and astrocytes, which is perfectly acceptable if the caveats of isolated cell systems are kept in mind. The paper would be considerably improved by investigation of these effect in Down Syndrome brain tissues, as indicated in my comments to the authors.

Referee #2 (Other Remarks):

Pierrot et al provide a very intriguing evaluation of the role of APP as a regulator of cholesterol homeostasis in cultured neurons. In rat cortical neurons, APP interacts with SREBP1 in the Golgi. APP overexpression was found to decrease cholesterol biosynthesis and SREBP1 and SREBP2 levels, whereas knockdown of APP expression increased cholesterol biosynthesis and SREBP1 and SREBP2 levels. The interactions and affect on cholesterol biosynthesis are due to the juxtamembrane domain in APP and do not occur in astrocytes. The major physiological impact of this interaction in neurons is suppression of the isoprenoid pathway that inhibits synaptic activity rather than cholesterol biosynthesis per se.

The data presented are very high quality and support the conclusions of the manuscript. The manuscript addresses a key question in the field and offers timely and novel information. There are, however, a few points that need to be addressed to enable acceptance of the manuscript for publication in EMM.

Major questions:

1. As SREBP1 controls de novo lipogenic genes, does the interaction of APP with SREBP1 affect fatty acid levels or composition in cultured neurons?

2. What proportion of total SREBP1 is in the Golgi to begin with? Is this an artifact of the culture conditions, ie movement of SREBP1 from ER to Golgi induced upon overexpression or knockdown procedures?

3. Please provide data on the specificity of the shRNA to APP and not APLP1/2.

4. Fig 2M does not provide convincing evidence of SCAP localization within the APP/SREBP1 complex.

5. Down Syndrome subjects inherit an extra copy of APP. Although there are conflicting reports about whether this leads to overexpression of APP protein in DS brain, in vivo relevance of the manuscript would be considerably augmented if these findings could be confirmed in DS brain tissues or neurons from DS subjects.

6. Does overexpression of APLP1/2 affect cholesterol biosynthesis and/or SREBP interactions in neurons? Does the Swedish mutation, which is close to the membrane spanning region of hAPP, exacerbate these effects on SREBP1 and cholesterol homeostasis?

Referee #3 (Comments on Novelty/Model System):

The involvement of APP in cellular cholesterol regultion has been demonstrated previously. Additional controls/experiments would be required to support the mechanism proposed by the authors

Referee #3 (Other Remarks):

Pierrot et al. describe effects of APP on cholesterol metabolism of primary neurons and astrocytes. Transduction of rat cortical neurons with human APP resulted in decreased mRNA expression of HMG-CoA reductase and reduced cholesterol biosynthesis without changing the steady-state cholesterol level. mRNA SREBP1 and 2 were also decreased upon APP expression, while shRNA mediated knock-down increased SREBP1 expression. Surprisingly, the inhibition of g-secretase and thus, inhibition of Abeta production, or a deletion construct lacking the APP ICD showed similar effects, suggesting that the observed effects on cholesterol biosynthesis and gene expression are independent of Abeta or AICD levels. Results from immunocytochemical and co-immunoprecipitation experiments suggested an interaction of APP with SREBP1, and authors speculate that APP inhibits cleavage of SREBP1 and translocation to the nucleus. These effects seem to be specific for neurons, because APP expression did not induce similar changes in primary astrocytes. Consistent with previous data, expression of APP impaired neuronal calcium oscillations. Impaired Ca^{2+} oscillations could also be mimicked by treatment with mevastatin, and rescued by addition of geranylgeraniol to APP overexpressing neurons. In brain slice cultures of APP transgenic mice, inhibiton of LTP could be partially recued by geranylgeraniol.

This is an interesting study linking APP to the neuronal cholesterol biosynthesis and isoprenoid metabolism. A major concern is the specificity of the observed effects for (human) APP. Further, in light of previous studies that also demonstrated a role of APP or its derivatives in cholesterol metabolism, the proposed mechanism in this study, direct interaction of APP and SREBP1, requires further proof.

Specific comments:

1. The controls using bgal encoding virus on cholesterol biosynthesis and expression of metabolic enzymes are not sufficient (fig. 1). The effects of APP should be compared to other membrane proteins also transported in the secretory pathway (e.g. the vesicular stomatitis virus glycoprotein and other members of the APP family, APLP1, APLP2). This is particular relevant as authors claim that other metabolites of APP, like Abeta, c-terminal fragments or AICD that have been proposed in previous studies to mediate the effects on cholesterol synthesis (Grimm et al., 2005, NCB; Tamboli et al., 2008 J.Neurosci; Liu et al., 2007 Neuron) were not involved in the observed effects. Does overexpression of mouse APP exert similar effects as human APP ? It has been shown previously that the proteolytic processing of mouse and human APP differs considerably in primary neurons (DeStrooper et al., EMBO J., 1995).

2. In addition to the determination of total cholesterol by enzymatic assays, the distribution of cellular cholesterol should also be visualized by cell staining with filipin in living neurons.

3. shRNA mediated knock-down of APP increased SREBP1 expression, MHGCoAR and cholesterol synthesis by about 100% (Fig. 1l-o). As shown in suppl. fig. 2, APP KO neurons have only about 30% increased levels of cholesterol as compared to APP+/+ cells. Authors should comment on the differential effects of knock-down as compared to knock-out.

4. The western blot and mRNA data (fig.1 and 2) demonstrate strong reduction of SREBP1 expression by overexpression of APP. Thus, it is not surprising that nuclear levels of SREBP1 are also lowered. However, on the images provided in fig. 2c-k SREBP1 reactivity is specifically reduced in the nucleus, but remained abundant in ER and/or Golgi compartments also in APP overexpressing cells. Authors explain this by a 'cytoplasmic retention'. However, when it would only be retention of SREBP1 in the ER/Golgi one would not expect such strong reductions in total SREBP1 as observed by western blotting. Authors should provide explanation for these discrepancies.

5. In the coIP experiments, the interaction of SREBP1 and SCAP appears to be reduced in neurons expressing hAPP or deletion constructs (C99, APPDC). What is the explanation for this effect ? Does APP compete with SCAP for binding to SREBP1 ?

6. A direct interaction of APP and SREBP1 should be proven by using additional mutations of APP in the Abeta and transmembrane domains for coIP experiments. This might also help to identify critical amino acids in APP that mediate binding. A recent study identified critical amino acids in the N-terminal portion of the Abeta domain of C99 for direct binding to cholesterol (Barrett et al., 2012, Science).

7. Levels of ATP6 also seem to be reduced in hAPP expressing cells (fig. 3d). Authors should provide an explaination.

8. The data on Ca2+ oscillations and LTP in cultured neurons and brain slices are interesting and one would expect from these results that knock-out of APP might improve LTP. However, it remains unclear how these findings relate to studies with APP knock-out mice, showing impaired LTP (Weyer et al., EMBO J, 2011). Authors should discuss these data and prove an explanation for the different effects between this and the previous study.

Resubmission 26 October 2012

Editor

EMBO Molecular Medicine

Dear Editor,

In response to your e-mail from August 8, 2012, we have now collected additional data, and decided to resubmit to EMBO Molecular Medicine, as you suggested.

All specific comments of the reviewers, except one related to LTP, concerned the biochemical data obtained in vitro, including analysis of calcium oscillations. Additional results that we present in the new version of the manuscript allowed us to strengthen these biochemical data. In the previous version of the manuscript, the last figure was indeed related to LTP analysis on brain slices. These electrophysiological analyses are still going on, and we are collecting data in WT, APP knockout and APP transgenic mice, using animals at different ages. At 9 month of age, we were able to reproducibly detect LTP deficits, but we have still to enlarge our number of samples and we found more appropriate to dissociate these results from the biochemical data presented in the new version of the manuscript.

Please find here below the detailed response to each reviewer comment.

Response to the reviewers' comments

Referee #1:

Generally speaking, this is an interesting and well written manuscript with sound biochemical data. It provides convincing evidence that APP expression affects cholesterol turnover via sterol regulatory element binding proteins (SREBP) in neurons. Basically, all the experiments have been done in two different ways (overexpression of human APP and APP knockout) to prove the role of APP in cholesterol biosynthesis. The biochemical results are very convincing as the co-localization of APP and SREBP1 in the Golgi and the identification of a possible interaction domain of APP with SERBP1. However, the important link between the biochemical observations and synaptic activity is not convincingly supported by the data presented: The demonstration that geranylgeraniol, an end product of the mevalonate pathway, rescues defects in calcium oscillations in cultured rat cortical neurons expressing human APP, as well as LTP defects in 5xFAD transgenic mice is by no means sufficient to convince this reviewer that APP and its proposed function in the cholesterol turnover is indeed crucial for synaptic function. An inhibition of the mevalonate pathway by APP may for example simply affect the resting membrane potential of neurons. A detailed electrophysiological analysis is needed to support the conclusions drawn by the authors.

We collected a lot of electrophysiological data on neurons expressing APP, which were previously published in The Journal of Neuroscience, 2009, 29(15):4708–4718. In this paper, we demonstrate that the resting potential of APP expressing neurons is not affected (Figure 2B). In APP expressing neurons, we measured a significant increase in L-type calcium currents (Figure 4D). The resulting calcium influx activated calcium-dependent potassium channels (SK Channels), increasing the pic

amplitude of medium after hyper polarization in APP expressing neurons (Figure 2B). Apamin, a specific antagonist of SK channels, was able to rescue calcium oscillations in APP expressing neurons (Figure 3C).

Results presented in the figure 7 of the new manuscript indicate that calcium oscillations inhibited by mevastatin are also rescued by 200 nM apamin, demonstrating that SK channels are also involved in the inhibition of calcium oscillations induced by alteration of cholesterol turnover. It was recently demonstrated (Campia et al., 2012) that enhancing the activity of the mevalonate pathway in cardiomyocytes increases the cellular levels of ubiquinone, and the synthesis of ATP, which can inhibit SK channels (Jiang and Haddad, 1997). A decrease in ATP, resulting from inhibition of the mevalonate pathway, could significantly activate SK channels, which have to be inhibited by apamin to rescue calcium oscillations. Interestingly, our preliminary data indicate a significant decrease in ATP in hAPP expressing neurons.

Referee #2 (Comments on Novelty/Model System):

The bulk of the manuscript uses cultured neurons and astrocytes, which is perfectly acceptable if the caveats of isolated cell systems are kept in mind. The paper would be considerably improved by investigation of these effect in Down Syndrome brain tissues, as indicated in my comments to the authors.

Referee #2 (Other Remarks):

Pierrot et al provide a very intriguing evaluation of the role of APP as a regulator of cholesterol homeostasis in cultured neurons. In rat cortical neurons, APP interacts with SREBP1 in the Golgi. APP overexpression was found to decrease cholesterol biosynthesis and SREBP1 and SREBP2 levels, whereas knockdown of APP expression increased cholesterol biosynthesis and SREBP1 and SREBP2 levels. The interactions and affect on cholesterol biosynthesis are due to the juxtamembrane domain in APP and do not occur in astrocytes. The major physiological impact of this interaction in neurons is suppression of the isoprenoid pathway that inhibits synaptic activity rather than cholesterol biosynthesis per se.

The data presented are very high quality and support the conclusions of the manuscript. The manuscript addresses a key question in the field and offers timely and novel information. There are, however, a few points that need to be addressed to enable acceptance of the manuscript for publication in EMM.

Major questions:

1. As SREBP1 controls de novo lipogenic genes, does the interaction of APP with SREBP1 affect fatty acid levels or composition in cultured neurons?

   The interaction of APP with SREBP1 not only affected de novo synthesis of cholesterol, but a 45 ± 4 % decrease in fatty acid levels was also measured in APP expressing neurons compared to control non-infected cells. This result has been added in the Supporting information Fig.1.

2. What proportion of total SREBP1 is in the Golgi to begin with? Is this an artifact of the culture conditions, ie movement of SREBP1 from ER to Golgi induced upon overexpression or knockdown procedures?

   In control neurons, immunofluorescence using an anti-N-terminal SREBP1 antibody clearly indicates a nuclear labeling, suggesting that the proportion of SREBP1 in the Golgi is low. Although SREBP1 co-localized with a Golgi marker in APP expressing neurons, a nuclear localization was observed upon knockdown of endogenous APP expression, confirming biochemical analysis of nuclear fractions. Therefore we cannot conclude that movement of SREBP1 from ER to Golgi is induced upon overexpression or knockdown procedures, and is related to an artifact of the culture conditions.

3. Please provide data on the specificity of the shRNA to APP and not APLP1/2.

   As shown in the figure 1 of the new manuscript, APP shRNA did not affect amounts of APLP1 and APLP2 detected in Western blotting, indicating specificity of the shRNA to APP and not APLP1/2.

4. Fig 2M does not provide convincing evidence of SCAP localization within the APP/SREBP1 complex.

   More convincing western blotting are now presented in figure 2L and M, in which SCAP is more clearly detected.

5. Down Syndrome subjects inherit an extra copy of APP. Although there are conflicting reports about whether this leads to overexpression of APP protein in DS brain, in vivo relevance of the manuscript would be considerably augmented if these findings could be confirmed in DS brain tissues or neurons from DS subjects.

   Although we didn't have any DS brain tissue available, we had the chance to analyze brain tissue from a familial AD cases with chromosome 21 micro duplication of the APP locus (Figures 6D and E added in the new manuscript). We think that down regulation of SREBP1 observed in that tissue, both at the mRNA and protein level is even more relevant than that we would have may be observed in DS. In DS, all the genes located on chromosome 21 are present in 3 copies, and overexpression of some of these genes could alter cholesterol homeostasis, independently from APP. In the familial AD case that we have analyzed, micro duplication of chromosome 21 is narrowed to the APP gene.

6. Does overexpression of APLP1/2 affect cholesterol biosynthesis and/or SREBP interactions in neurons? Does the Swedish mutation, which is close to the membrane spanning region of hAPP, exacerbate these effects on SREBP1 and cholesterol homeostasis?

   Although we didn't express APLP1 or APLP2 in cortical neurons, we observed that up regulation of SREBP1 expression in APP knockout neurons was much less important than that observed following treatment of control neurons with APP shRNA. In the supplemental figure 3 of the new manuscript, we demonstrate a significant up regulation of APLP1 in APP knockout neurons that we did not observed in APP shRNA treated neuron (new Figure 1), suggesting partial compensation by APLP1.

   In the figure 1 of the new manuscript, we show that expression of Swedish APP can also down regulate both HMGCR and SREBP, but without exacerbation as compared to wild type APP. This further argues for an Abeta-independent effect.

Referee #3 (Comments on Novelty/Model System):

The involvement of APP in cellular cholesterol regultion has been demonstrated previously. Additional controls/experiments would be required to support the mechanism proposed by the authors

Referee #3 (Other Remarks):

Pierrot et al. describe effects of APP on cholesterol metabolism of primary neurons and astrocytes. Transduction of rat cortical neurons with human APP resulted in decreased mRNA expression of HMG-CoA reductase and reduced cholesterol biosynthesis without changing the steady-state cholesterol level. mRNA SREBP1 and 2 were also decreased upon APP expression, while shRNA mediated knock-down increased SREBP1 expression. Surprisingly, the inhibition of g-secretase and thus, inhibition of Abeta production, or a deletion construct lacking the APP ICD showed similar effects, suggesting that the observed effects on cholesterol biosynthesis and gene expression are independent of Abeta or AICD levels. Results from immunocytochemical and co-immunoprecipitation experiments suggested an interaction of APP with SREBP1, and authors speculate that APP inhibits cleavage of SREBP1 and translocation to the nucleus. These effects seem to be specific for neurons, because APP expression did not induce similar changes in primary astrocytes. Consistent with previous data, expression of APP impaired neuronal calcium oscillations. Impaired Ca2+ oscillations could also be mimicked by treatment with mevastatin, and rescued by addition of geranylgeraniol to APP overexpressing neurons. In brain slice cultures of APP transgenic mice, inhibiton of LTP could be partially recued by geranylgeraniol.

This is an interesting study linking APP to the neuronal cholesterol biosynthesis and isoprenoid metabolism. A major concern is the specificity of the observed effects for (human) APP. Further, in light of previous studies that also demonstrated a role of APP or its derivatives in cholesterol metabolism, the proposed mechanism in this study, direct interaction of APP and SREBP1, requires further proof.

Specific comments:

1. The controls using bgal encoding virus on cholesterol biosynthesis and expression of metabolic enzymes are not sufficient (fig. 1). The effects of APP should be compared to other membrane proteins also transported in the secretory pathway (e.g. the vesicular stomatitis virus glycoprotein and other members of the APP family, APLP1, APLP2). This is particular relevant as authors claim that other metabolites of APP, like Abeta, c-terminal fragments or AICD that have been proposed in previous studies to mediate the effects on cholesterol synthesis (Grimm et al., 2005, NCB; Tamboli et al., 2008 J.Neurosci; Liu et al., 2007 Neuron) were not involved in the observed effects. Does overexpression of mouse APP exert similar effects as human APP ? It has been shown previously that the proteolytic processing of mouse and human APP differs considerably in primary neurons (DeStrooper et al., EMBO J., 1995).

   The best negative control that we could imagine is related to point 6 of the same reviewer. In co-immunoprecipitation experiments, we demonstrate in Figure 3 that C99 is able to interact with SREBP1. As mentioned by the reviewer, a recent study identified critical amino acids in the C99 for direct binding to cholesterol (Barrett et al., 2012). We therefore decided to express C99 mutated in the GxxxG motif to investigate whether this sequence reported to be involved in direct binding to cholesterol would be important for control of cholesterol synthesis. Interestingly, we were unable to detect interaction between SREBP1 and mutated C99. In addition, in the Figure 3 of the new version of the manuscript, we demonstrate that for similar expression levels, C99, but no mutated C99, is able to down regulate HMGCR mRNA levels. C99 mutated in the GxxxG motif therefore becomes the best negative control.

   We completely agree that the proteolytic processing of mouse and human APP differs considerably in primary neurons. However, down regulation of endogenous mouse APP clearly shows opposite effects as compared to overexpression of human APP, indicating that APP, either endogenous (mouse) or overexpressed (human) can control cholesterol turnover.

2. In addition to the determination of total cholesterol by enzymatic assays, the distribution of cellular cholesterol should also be visualized by cell staining with filipin in living neurons.

Control neurons *APP expressing neurons*

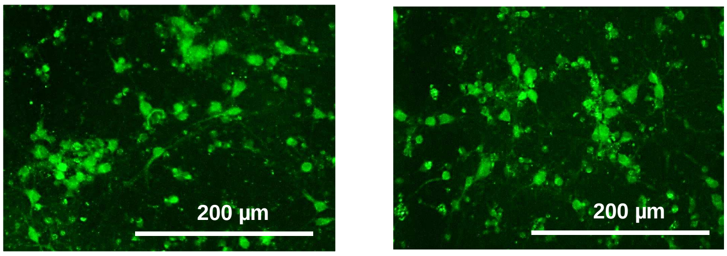

In living control and APP expressing neurons, filipin staining confirms results presented in figure 7 of the new manuscript showing that APP does not modify total cholesterol content.

3. shRNA mediated knock-down of APP increased SREBP1 expression, MHGCoAR and cholesterol synthesis by about 100% (Fig. 1l-o). As shown in suppl. fig. 2, APP KO neurons have only about 30% increased levels of cholesterol as compared to APP+/+ cells. Authors should comment on the differential effects of knock-down as compared to knock-out.

   In the supplemental Figure 3 of the new manuscript, we demonstrate a significant up regulation of APLP1 in APP knockout neurons that we did not observed in APP shRNA treated neurons (new Figure 1). Therefore, a partial compensation by APLP1 in APP knockout neurons could explain the differential effects of knock-down as compared to knock-out.

4. The western blot and mRNA data (fig.1 and 2) demonstrate strong reduction of SREBP1 expression by overexpression of APP. Thus, it is not surprising that nuclear levels of SREBP1 are also lowered. However, on the images provided in fig. 2c-k SREBP1 reactivity is specifically reduced in the nucleus, but remained abundant in ER and/or Golgi compartments also in APP overexpressing cells. Authors explain this by a 'cytoplasmic retention'. However, when it would only be retention of SREBP1 in the ER/Golgi one would not expect such strong reductions in total SREBP1 as observed by western blotting. Authors should provide explanation for these discrepancies.

   Contrary to immunofluorescence, Western blotting is much more quantitative. We agree with the reviewer that 'cytoplasmic retention' suggests no modification of global synthesis and is therefore inappropriate. Our data indicate that in addition to a decreased synthesis, SREBP1 is localized into the Golgi and not in the nucleus. This has been corrected in the new version of our manuscript.

5. In the coIP experiments, the interaction of SREBP1 and SCAP appears to be reduced in neurons expressing hAPP or deletion constructs (C99, APPDC). What is the explanation for this effect ? Does APP compete with SCAP for binding to SREBP1 ?

In cellular lysates from APP expressing neurons, SREBP1 protein levels are decreased (figure 1). In neurons expressing C99 or APP deleted in its C-terminal domain (APP△C), SREBP1 protein levels are decreased to similar extent (see figure below). In coIP experiments (figure 3), the input shows a decrease in SREBP1 protein levels in neurons expressing APP, APP△C and C99. Although stoichiometric interaction between SREBP1 and SCAP was never demonstrated, a reduction of SCAP in the immune precipitate with SREBP1 does not probably result from competition between APP and SCAP for binding to SREBP1, but rather from a decrease in SREBP1 in neurons expressing APP, APP△C and C99.

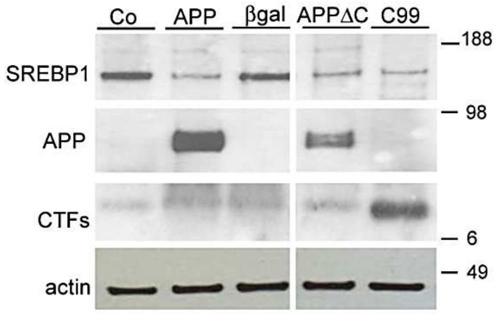

6. A direct interaction of APP and SREBP1 should be proven by using additional mutations of APP in the Abeta and transmembrane domains for coIP experiments. This might also help to identify critical amino acids in APP that mediate binding. A recent study identified critical amino acids in the N-terminal portion of the Abeta domain of C99 for direct binding to cholesterol (Barrett et al., 2012, Science).

   Thank You for this comment, allowing us to express a C99 mutant that became the best negative control (see point 1).

7. Levels of ATP6 also seem to be reduced in hAPP expressing cells (fig. 3d). Authors should provide an explaination.

   It was previously demonstrated (Zeng et al., 2004) that SREBP2, by recruiting HDAC1 to the ATF6-SREBP2 complex, is able to antagonize SREBP2. Therefore, APP and ATF6 could both inhibit SREBP2 by different ways. Consequently, down regulation of ATF6 expression by hAPP could be considered as a retro control of SREBP2 target genes expression.

8. The data on Ca2+ oscillations and LTP in cultured neurons and brain slices are interesting and one would expect from these results that knock-out of APP might improve LTP. However, it remains unclear how these findings relate to studies with APP knock-out mice, showing impaired LTP (Weyer *et al.*, 2011). Authors should discuss these data and prove an explanation for the different effects between this and the previous study.

   In cultured neurons, down regulation of endogenous APP using shRNA increases the frequency of calcium oscillations, but also significantly decreases their amplitude.

Therefore, we would not expect that knockout of APP might improve LTP. Since we are still investigating APP transgenic animals as well as APP knockout animals, and are waiting for enough old animals in which LTP deficits can be observed, we found more appropriate to dissociate these results from the biochemical data presented in the new version of the manuscript.

REFERENCES

1. *Barrett PJ, Song Y, Van Horn WD, Hustedt EJ, Schafer JM, Hadziselimovic A, Beel AJ, and Sanders CR (2012) The amyloid precursor protein has a flexible transmembrane domain and binds cholesterol. Science, 336, 1168-1171.*

2. *Campia I, Sala V, Kopecka J, Leo C, Mitro N, Costamagna C, Caruso D, Pescarmona G, Crepaldi T, Ghigo D, Bosia A, and Riganti C (2012) Digoxin and ouabain induce the efflux of cholesterol via liver X receptor signalling and the synthesis of ATP in cardiomyocytes. Biochem J, 447, 301-311.*

3. *Jiang C and Haddad GG (1997) Modulation of K+ channels by intracellular ATP in human neocortical neurons. J Neurophysiol, 77, 93-102.*

4. *Weyer SW, Klevanski M, Delekate A, Voikar V, Aydin D, Hick M, Filippov M, Drost N, Schaller KL, Saar M, Vogt MA, Gass P, Samanta A, Jaschke A, Korte M, Wolfer DP, Caldwell JH, and Muller UC (2011) APP and APLP2 are essential at PNS and CNS synapses for transmission, spatial learning and LTP. EMBO J, 30, 2266-2280.*

5. *Zeng L, Lu M, Mori K, Luo S, Lee AS, Zhu Y, and Shyy JY (2004) ATF6 modulates SREBP2-mediated lipogenesis. EMBO J, 23, 950-958.*

2nd Editorial Decision 17 December 2012

Thank you for the submission of your manuscript to EMBO Molecular Medicine. We have now heard back from the two referees whom we asked to evaluate your manuscript. As you will see from the reports below, the reviewers acknowledge the relevance and interest of your work.

However, reviewer #2 (this is reviewer #3 of your previous submission) raises significant concerns. We would be open to consider a revised manuscript that convincingly addresses all the issues raised by experimentation where appropriate.

Reviewer #2 is concerned about the specificity of the observed effects of APP on SREBP1 and cholesterol. Importantly, s/he suggests analyzing the effects of stabilising full-length APP and would like to see further, rigorous Co-IP experiments to substantiate the proposed mechanism. This reviewer also requires analysis of the effects of full-length APP carrying the G625/G629L mutations on cholesterol biosynthesis and interaction with SREBP1. Reviewer #2 further suggests directly comparing the effects of APP and APLPs. In addition, s/he would like to see additional data to substantiate the link between the effects of APP on calcium oscillations and the observed changes in cholesterol metabolism.

We have consulted an additional expert advisor to comment on your revised manuscript and the reviewers' comments. The advisor acknowledges the potential interest of your study, but agrees with the concerns of reviewer #2 and recommends addressing all of this reviewer's concerns. We would therefore be willing to consider a revised manuscript with the understanding that the referee concerns must be fully addressed.

Please also change the title and abstract according to reviewer #1's suggestion.

Please note that it is EMBO Molecular Medicine policy to allow a single round of revision in order to avoid the delayed publication of research findings. Consequently, acceptance or rejection of the manuscript will depend on the completeness of your responses included in the next version of the manuscript.

EMBO Molecular Medicine has a "scooping protection" policy, whereby similar findings that are published by others during review or revision are not a criterion for rejection. I understand that the amount of work that would be required to submit a revised version of your manuscript is significant, hence, should you decide to submit a revised version, I do ask that you get in touch after three months if you have not completed it, to update us on the status.

Please also contact us as soon as possible if similar work is published elsewhere, as we then may not be able to extend the revision period beyond three months.

***** Reviewer's comments *****

Referee #1 (General Remarks):

The author's addressed my point that the biochemical data are not convincingly supported by electrophysiological LTP studies in brain slices and decided to dissociate these data from the manuscript what I highly appreciate. However they did not change the title and the abstract accordingly. Synaptic responses or currents were not analysed in this study but neuronal activity only. Therefore the title and the conclusion in the abstract that their findings are important for synaptic activity are very misleading and still have to be changed.

Referee #2 (General Remarks):

In the revised manuscript, Pierrot et al. further addressed the role of APP in neuronal cholesterol metabolism. Overexpression of human APP decreases de novo synthesis of cholesterol without changing the steady state levels in primary rat cortical neurons, but not in primary astrocytes. In neurons, overexpression of APP also decreased expression of SREBP1 at the mRNA and protein level. mRNA of HMGCoAR was also reduced. Conversely, knock-down of endogenous APP increased cholesterol synthesis and SREBP1 expression.

Co-immunoprecipitations indicate an interaction of APP with SREBP1. Authors now show in addition that the C99 c-terminal fragment of APP also co-precipitates with SREBP1, and C99 overexpression decreased levels of SREBP1 and HMGCoAR. Glycine mutants of C99 recently shown to affect the direct interaction with cholesterol did not decrease HMGCoAR mRNA levels, suggesting a role of these residues in the interaction with SREBP1. Authors also include additional data from human brains showing an inverse correlation of APP and SREBP1 expression.

Together, the study convincingly demonstrates the involvement of APP in neuronal cholesterol metabolism. The conclusion of the authors that the observed effects are mediated by full-length APP is, however, not fully supported by the present data. It also remains unclear whether other members of the APP family, APLP1 and APLP2, might also regulate cholesterol biosynthesis. As compared to the previous version of the manuscript, authors now focussed more on the biochemical analysis of APP effects on SREBP1 and cholesterol. However, the new data also raise additional questions about the specificity of the observed effects that require further experiments to strengthen the conclusion of the authors. The relevance of the findings related to synaptic activity also remains unclear. Further attempts are required to support an involvement of cholesterol metabolites in the APP dependent suppression of calcium oscillations.

Specific comments:

1. Authors make a strong case that the observed effects on HMGCoAR expression are caused by full-length APP and in a gamma-secretase independent manner. This is not fully clear from the experimental approaches. Authors show that C99 exerts similar effects (Fig. 3), and APP CTFs were also increased upon overexpression of full-length APP (fig. 1f).

That cell treatment with DAPT did not further decrease HMGCoAR expression, is not a proof that the observed effects are independent of gamma-secretase activity. A decrease by 70-80 % in HMGCoAR mRNA expression by overexpressed full-length APP (fig. 1g) is already very strong, and might have reached a maximum. Thus, additional increase in APP C99 levels by DAPT treatment might not exert additive effects.

The relative contribution of overexpressed full-length APP or derived CTFs remains to be determined.

Authors should stabilize full-length APP by inhibition of alpha- and beta-secretase and then analyze effects on cholesterol biosynthesis, SREBP1 and HMGCoAR.

2. Along the same line, co-immunoprecipitation experiments are not rigorous and raise some additional questions.
- Does the APP C99 also co-immunoprecipitate with SREBP1 and vice-a-versa ? Precipitates with the anti-SREBP1 antibody (fig. 2 m,n) should also be tested for APP CTF.

- Does APP also interact with SCAP ? Precipitates with anti-APP antibodies (fig.2n) should also be tested for SCAP (as was done for SREBP co-IPs in fig. 2m).

- Authors write that they "were unable to co-immunoprecipitate SREBP1 with C99 G625L/G629L mutant",... These data are not shown in the manuscript. Authors should perform co-IP studies for C99 wild-type and C99 G625L/G629L in parallel and test for co-precipitation of SREBP and SCAP.

- How would a lack of cholesterol interaction with the C99 G625L/G629L mutant affect the interaction with SREBP1 ? Is this interaction dependent on cholesterol ? This would be surprising as

SREBP1 itself has no known cholesterol sensing domain, in contrast to SCAP.

- Overall, it is somehow surprising that authors only use the C99 construct to test the effect on HMGCoAR expression, when their main point is the role of full-length APP in cholesterol regulation. To strengthen this conclusion it would be much more compelling to introduce the G625L/G629L mutations in the full-length APP and test the effect on cholesterol biosynthesis and interaction with SREBP1 (and SCAP).

3. Another main point is the specificity of the observed effects for APP. Authors show that knock-down of endogenous APP did not affect levels of APLP1 and APLP2 and found an approximately two fold increase in HMGCoAR mRNA, SREBP1 protein levels and cholesterol biosynthesis (fig.1 l-o), leading to the speculation that APLPs only play a minor if any role in SREBP1 regulation. However, in the supplement (fig. S3), APP-/- cells showed a much weaker effect on SREBP1 levels (increase by 30%), and authors interpret this by a partial compensation by upregulated APLP1.
To directly compare the effects of APP and APLPs, authors should use the proteins in the same experimental paradigm, e.g. overexpression in primary neurons. This would also be a very good control for the role of the GXXXG motif in APP, as it is not conserved in the APLPs.

- Does APLP1 and 2 also co-precipitate with SREBP1 ?

4. The functional data on APP dependent synaptic activity confirm previous studies of the authors (fig. 7 i-k). However, the new experiments to link the effects of APP on neuronal calcium oscillations to the observed changes in cholesterol metabolism appear preliminary. What is shown in fig. 7l is that statin treatment of neuronal cultures mimics the effects of APP overexpression. Whether the APP dependent suppression of network activity indeed involves changes in the metabolites of the cholesterol and isoprenoid pathway, as discussed by the authors, requires further proof. Authors should design experiments to rescue suppressed calcium oscillations by supplementation with isoprenoid metabolites. Such experiments were included in the previous version of the manuscript, but have been removed in the revision.
This is an important point, because basal levels of cholesterol are not changed by APP overexpression in this experimental system.

Minor points:

Levels of (endogenous) mouse Abeta are much higher (490 pg/ml) than human Abeta from overexpressed APP (28 pg/ml) (fig. 1H). Authors should give an explanation.

1st Revision - authors' response 21 January 2013

In response to your e-mail from December 17, 2012, we have now collected additional data, and are able to resubmit a revised manuscript to EMBO Molecular Medicine.

You will appreciate that all specific comments of the reviewers have been addressed in the revised version of the manuscript. Figures 2, 3, and 7 have been modified, and additional data are provided in supporting information figures 6 and 7. Therefore, we hope that this revised manuscript will be suitable for publication in EMBO Molecular Medicine.

Please find here below the detailed response to each reviewer comment.

Referee #1 (General remarks):

The author's addressed my point that the biochemical data are not convincingly supported by electrophysiological LTP studies in brain slices and decided to dissociate these data from the manuscript what I highly appreciate. However they did not change the title and the abstract accordingly. Synaptic responses or currents were not analyzed in this study but neuronal activity only. Therefore the title and the conclusion in the abstract that their findings are important for synaptic activity are very misleading and still have to be changed.

According to the reviewer comment, "synaptic activity "has been replaced by "neuronal activity" in the manuscript. The revised manuscript is entitled "Amyloid Precursor Protein Controls Cholesterol Turnover Needed For Neuronal Activity".

Referee #2 (General remarks):

In the revised manuscript, Pierrot et al. further addressed the role of APP in neuronal cholesterol metabolism. Overexpression of human APP decreases de novo synthesis of cholesterol without changing the steady state levels in primary rat cortical neurons, but not in primary astrocytes. In neurons, overexpression of APP also decreased expression of SREBP1 at the mRNA and protein level. mRNA of HMGCoAR was also reduced. Conversely, knock-down of endogenous APP increased cholesterol synthesis and SREBP1 expression.

Co-immunoprecipitations indicate an interaction of APP with SREBP1. Authors now show in addition that the C99 c-terminal fragment of APP also co-precipitates with SREBP1, and C99 overexpression decreased levels of SREBP1 and HMGCoAR. Glycine mutants of C99 recently shown to affect the direct interaction with cholesterol did not decrease HMGCoAR mRNA levels, suggesting a role of these residues in the interaction with SREBP1. Authors also include additional data from human brains showing an inverse correlation of APP and SREBP1 expression.

Together, the study convincingly demonstrates the involvement of APP in neuronal cholesterol metabolism. The conclusion of the authors that the observed effects are mediated by full-length APP is, however, not fully supported by the present data. It also remains unclear whether other members of the APP family, APLP1 and APLP2, might also regulate cholesterol biosynthesis. As compared to the previous version of the manuscript, authors now focused more on the biochemical analysis of APP effects on SREBP1 and cholesterol. However, the new data also raise additional questions about the specificity of the observed effects that require further experiments to strengthen the conclusion of the authors. The relevance of the findings related to synaptic activity also remains unclear. Further attempts are required to support an involvement of cholesterol metabolites in the APP dependent suppression of calcium oscillations.

Specific comments:

1. Authors make a strong case that the observed effects on HMGCoAR expression are caused by full-length APP and in a gamma-secretase independent manner. This is not fully clear from the experimental approaches. Authors show that C99 exerts similar effects (Fig. 3), and APP CTFs were also increased upon overexpression of full-length APP (fig. 1f).

That cell treatment with DAPT did not further decrease HMGCoAR expression, is not a proof that the observed effects are independent of gamma-secretase activity. A decrease by 70-80 % in HMGCoAR mRNA expression by overexpressed full-length APP (fig. 1g) is already very strong, and might have reached a maximum. Thus, additional increase in APP C99 levels by DAPT treatment might not exert additive effects.

The relative contribution of overexpressed full-length APP or derived CTFs remains to be determined.

Authors should stabilize full-length APP by inhibition of alpha- and beta-secretase and then analyze effects on cholesterol biosynthesis, SREBP1 and HMGCoAR.

Although we investigated the role of full length APP on HMGCoAR expression, we conclude that full length APP, C99 and APP deleted from its C-terminal domain are able to regulate HMGCoAR expression. Therefore, we do not "make a strong case that the observed effects on HMGCoAR expression are caused by full-length APP". We agree that APP CTFs were also increased upon overexpression of full-length APP. In contrast, expression of APPDC, which cannot produce any CTFs, did not increase APP CTFs but still down regulated HMGCoAR expression.

Both CTFs and full length APP are substrates of gamma-secretase to release the intracellular domain AICD, which can regulate transcription of several genes. Following inhibition of the gamma-secretase by DAPT, we agree with the reviewer that additional increase in APP C99 levels might not exert additive decrease in HMGCoAR mRNA levels, because a decrease by 70-80 % in expression by overexpressed full-length APP is already very strong, and might have reached a maximum. However, we determined the relative contribution of overexpressed full-length APP or derived CTFs, not only by using DAPT, but also by expressing APPDC, which does not induce any

increase in APP C99 levels. Results presented in figure 1D and E clearly show that expression of APPDC down regulates HMGCR mRNA levels like APP, demonstrating that the intracellular C-terminal domain of APP is dispensable. This perfectly fits with results presented in figure 3A demonstrating that APP, APPDC and C99 interact with SREBP and down regulate HMGCR mRNA levels, indicating that the juxta- and transmembrane domain of APP plays a key role in this regulation.

2. Along the same line, co-immunoprecipitation experiments are not rigorous and raise some additional questions.

- Does the APP C99 also co-immunoprecipitate with SREBP1 and vice-a-versa ? Precipitates with the anti-SREBP1 antibody (fig. 2 m,n) should also be tested for APP CTF.

Results presented in the panel A of figure 3 from the previous version of the manuscript already demonstrated that C99 co-immunoprecipitates with SREBP1. If APP C99 corresponds to C99 produced following expression of APP, new co-immunoprecipitation experiments were performed and results presented in the new figure 2N indicate that C99 produced following expression of APP co-immunoprecipitates with SREBP1. In addition, in the new panel A of the supporting information figure 6, we demonstrate that endogenous APP CTF also co-immunoprecipitates with SREBP1.

- Does APP also interact with SCAP ? Precipitates with anti-APP antibodies (fig.2n) should also be tested for SCAP (as was done for SREBP co-IPs in fig. 2m).

New co-immunoprecipitation experiments were performed to address the issue of APP:SCAP interaction. Results presented in the new figure 2O indicate that SCAP co-immunoprecipitates with APP.

- Authors write that they "were unable to co-immunoprecipitate SREBP1 with C99 G625L/G629L mutant",... These data are not shown in the manuscript. Authors should perform co-IP studies for C99 wild-type and C99 G625L/G629L in parallel and test for co-precipitation of SREBP and SCAP.

Additional experiments were performed using the C99 G625L/G629L mutant. Results presented in the new figure 3C,D,E indicate that, contrary to C99, expression of this mutant does not modify both SREBP1 protein and HGMCR mRNA levels. In addition, C99 clearly co-immunoprecipitated with SREBP1, while C99 G625L/G629L mutant did not (new figure 3F).

- How would a lack of cholesterol interaction with the C99 G625L/G629L mutant affect the interaction with SREBP1 ? Is this interaction dependent on cholesterol ? This would be surprising as SREBP1 itself has no known cholesterol sensing domain, in contrast to SCAP.

We never claimed that APP:SREBP1 interaction is dependent on cholesterol. As mentioned in the paragraph entitled "A GXXXG motif of the transmembrane domain of APP is critical for SREBP1-mediated regulation of HMGCR expression", "our results indicate that a GXXXG motif of C99, predicted from structural analysis to be essential to cholesterol binding, is critical for SREBP1-mediated regulation of HMGCR expression". In addition, APP, under condition of excess or defect, did not modify neuronal membrane cholesterol content, as demonstrated in figure 7C. Structural analyses suggesting a possible interaction between C99 and cholesterol could argue for a possible role of APP as a cholesterol sensor. We mentioned in the discussion that "Whether, in addition to SCAP, APP might function as neuronal cholesterol sensors and/or cargo proteins required for shuttling SREBP1 between ER and Golgi deserves further investigations".

- Overall, it is somehow surprising that authors only use the C99 construct to test the effect on HMGCoAR expression, when their main point is the role of full-length APP in cholesterol regulation. To strengthen this conclusion it would be much more compelling to introduce the G625L/G629L mutations in the full-length APP and test the effect on cholesterol biosynthesis and interaction with SREBP1 (and SCAP).

The simple reason why we used the C99 construct rather than to introduce the G625L/G629L mutations in the full-length APP is that, as suggested by the reviewer, we worked in the conditions reported by Barrett et al. Science. 2012 (6085):1168-71. In this paper entitled "The amyloid precursor protein has a flexible transmembrane domain and binds cholesterol", structural analyses were performed on C99 and not on full length APP. In addition, following shedding of APP by beta-secretase, C99 perfectly mimics full length APP (Beel et al. Biochemistry. 2008 (47): 9428-46).

3. Another main point is the specificity of the observed effects for APP. Authors show that knock-down of endogenous APP did not affect levels of APLP1 and APLP2 and found an approximately two fold increase in HMGCoAR mRNA, SREBP1 protein levels and cholesterol biosynthesis (fig.1 l-o), leading to the speculation that APLPs only play a minor if any role in SREBP1 regulation. However, in the supplement (fig. S3), APP-/- cells showed a much weaker effect on SREBP1 levels (increase by 30%), and authors interpret this by a partial compensation by up regulated APLP1.

To directly compare the effects of APP and APLPs, authors should use the proteins in the same experimental paradigm, e.g. overexpression in primary neurons. This would also be a very good control for the role of the GXXXG motif in APP, as it is not conserved in the APLPs.

- Does APLP1 and 2 also co-precipitate with SREBP1 ?

As previously requested, we provided data on the specificity of the shRNA to APP and not APLP1/2, as shown in figure 1L. As previously mentioned by the reviewer, " APP KO neurons have only about 30% increased levels of cholesterol as compared to APP+/+ cells. Authors should comment on the differential effects of knock-down as compared to knock-out". We therefore provided additional data indicating that in APP knockout neurons, a very significant up regulation of APLP1 (150 ± 6 %, n=3) but not APLP2 was observed (Supporting information Fig. 3C). This up regulation of APLP1 expression in APP knockout neurons allows to reach APLP1 levels (1.5), very similar to those of APP (1.6) in APP expressing neuron (Fig.1A). These data do not lead to the speculation that APLPs, and in particular APLP1, only play a minor if any role in SREBP1 regulation.

In addition, new co-immunoprecipitation experiments were performed to address the issue of APLP1 and 2 co-precipitation with SREBP1. In the new supporting information Fig. 7, we show co-precipitation of APLP1 and APLP2 with SREBP1. Although the GXXXG motif in APP is not conserved as such in APLPs, it is interesting to note that the second G of the GXXXG motif is conserved in APLPs (Aydin et al. Exp Brain Res. 2012 (217): 423-34.) and that this residue is even more important than the first one for interaction with cholesterol, as demonstrated in the paper of Barrett et al. Science. 2012 (6085):1168-71.

4. The functional data on APP dependent synaptic activity confirm previous studies of the authors (fig. 7 i-k). However, the new experiments to link the effects of APP on neuronal calcium oscillations to the observed changes in cholesterol metabolism appear preliminary. What is shown in fig. 7l is that statin treatment of neuronal cultures mimics the effects of APP overexpression. Whether the APP dependent suppression of network activity indeed involves changes in the metabolites of the cholesterol and isoprenoid pathway, as discussed by the authors, requires further proof. Authors should design experiments to rescue suppressed calcium oscillations by supplementation with isoprenoid metabolites. Such experiments were included in the previous version of the manuscript, but have been removed in the revision.

This is an important point, because basal levels of cholesterol are not changed by APP overexpression in this experimental system.

According to the reviewer comments, the new figure 7 has been modified to include partial rescue of calcium oscillation by isoprenoid metabolite.

Minor points:

Levels of (endogenous) mouse Abeta are much higher (490 pg/ml) than human Abeta from overexpressed APP (28 pg/ml) (fig. 1H). Authors should give an explanation.

In cell lines, overexpression of human APP induces secretion of huge amounts of Abeta. In contrast, neuronal expression of human APP at similar levels induces the secretion of about 30 times less Abeta (Feyt et al. J Biol Chem. 2005 (280):33220-7). In addition, in the present study, overexpression of human APP only accounts for 60 % of endogenous APP, explaining why levels of (endogenous) mouse Abeta are much higher (490 pg/ml) than human Abeta from overexpressed APP (28 pg/ml).

Accepted 06 February 2013

The manuscript has been re-reviewed by one original referee, who had no further comments.
