# Supplementary figures and images for: Amyloid precursor protein controls cholesterol turnover needed for neuronal activity
Source: EMBO Mol Med. 2013 Apr 2;5(4):608–25. doi: 10.1002/emmm.201202215 (PMC3628100; doi:10.1002/emmm.201202215)

Fig. 1F

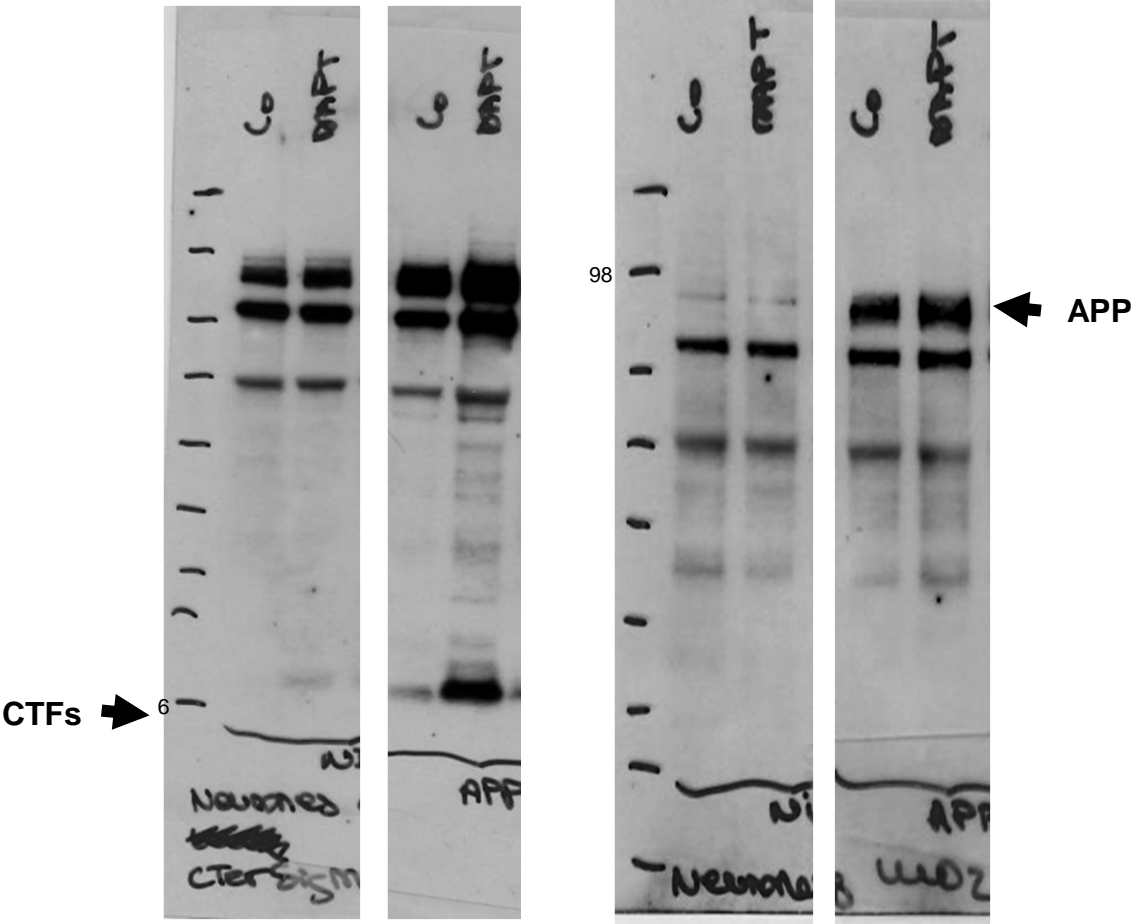

Fig. 1J

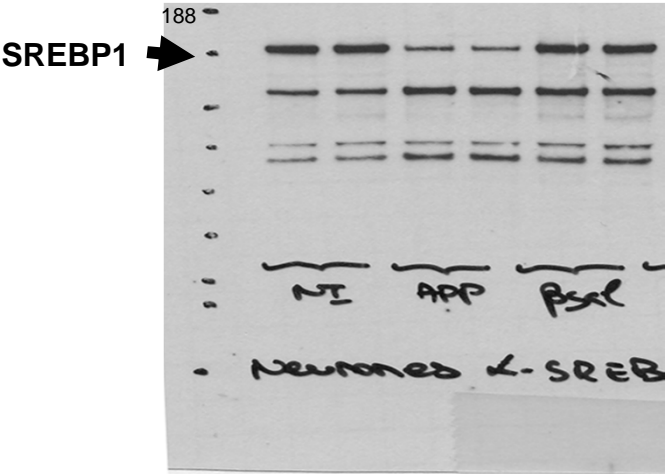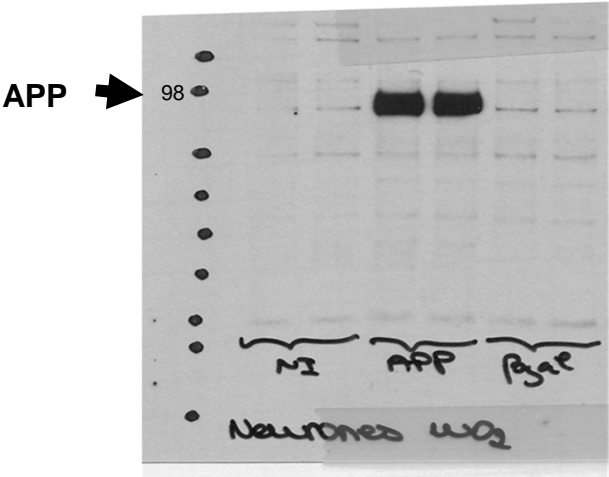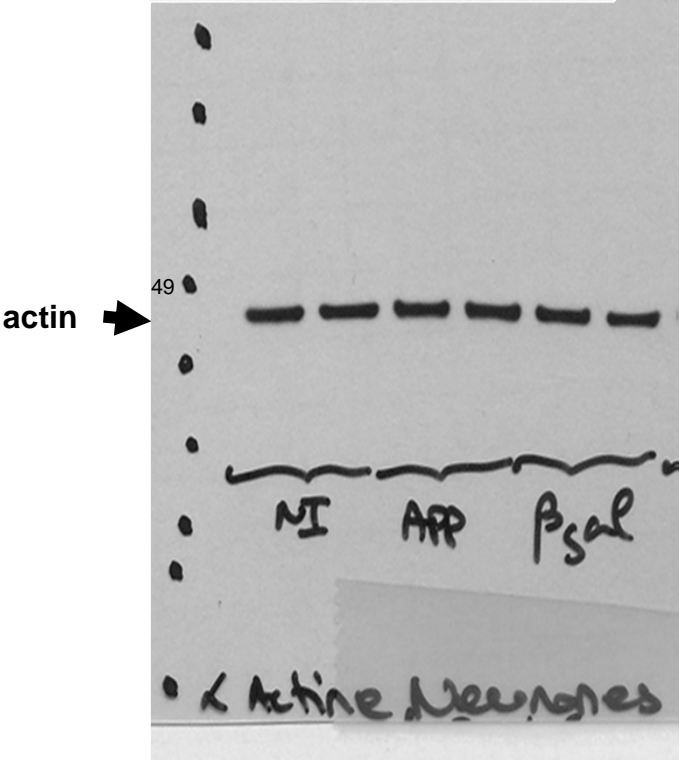

Fig. 1L

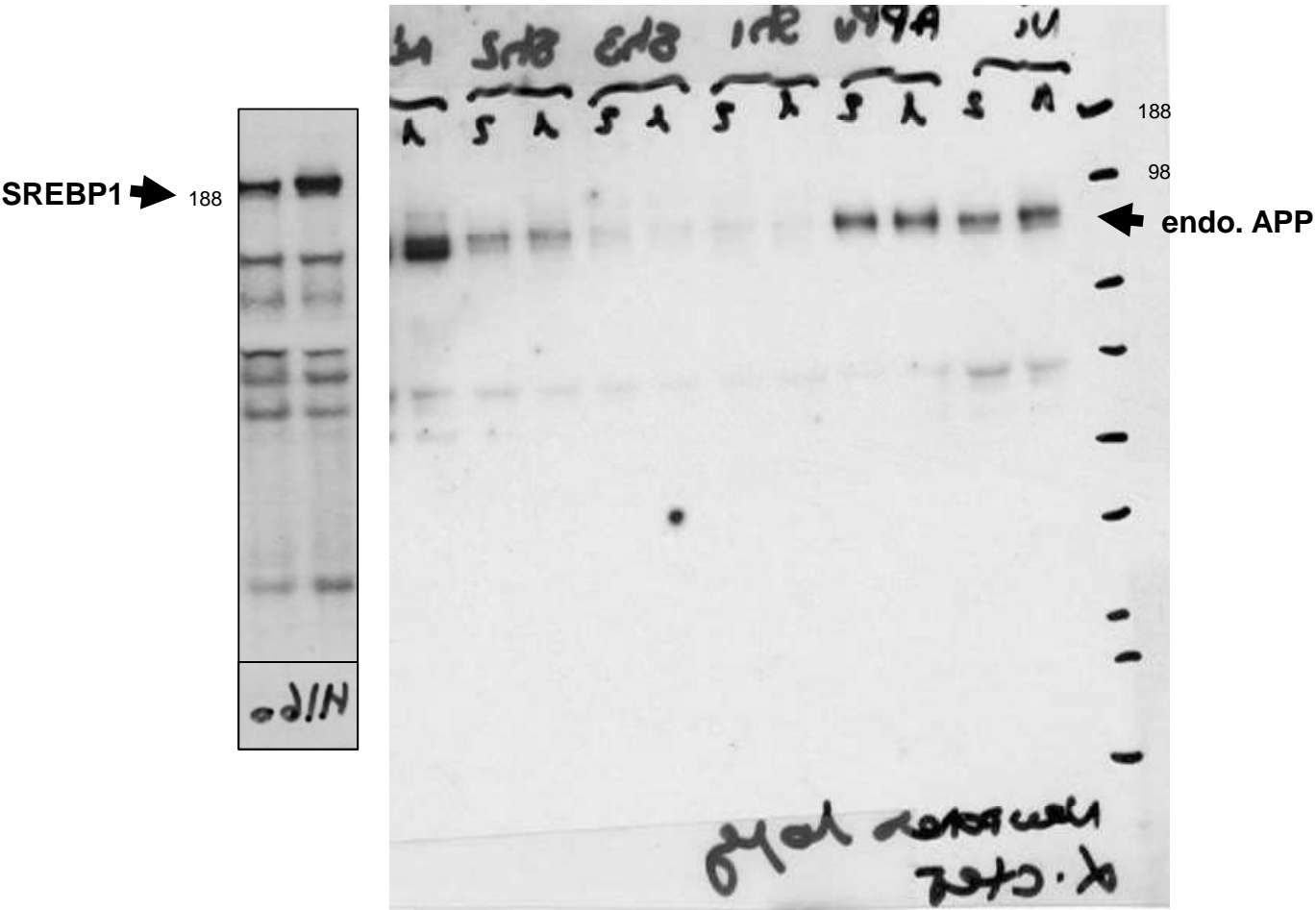

Supplement: Supplementary file 2 [file emmm0005-0608-sd2.pdf]

Fig. 2A

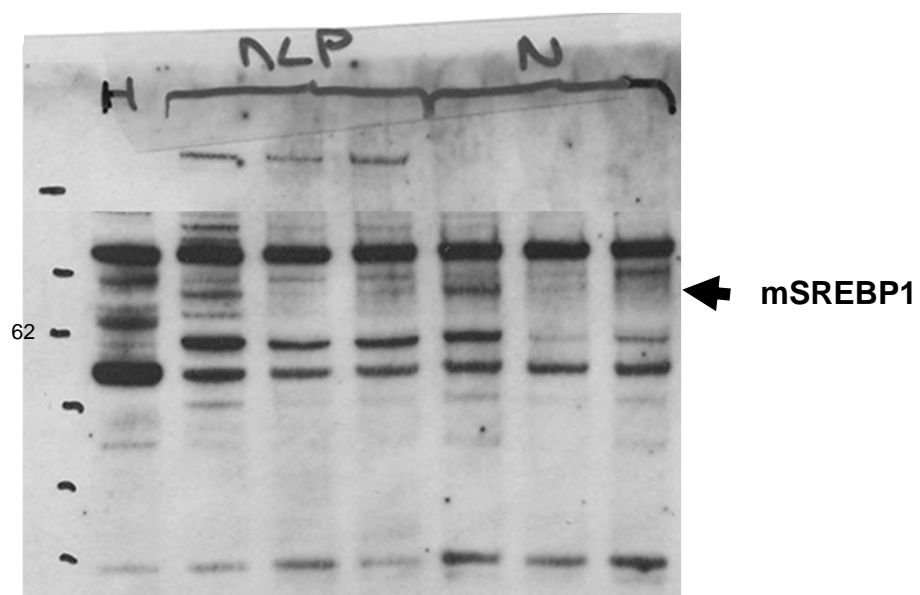

Fig. 2B

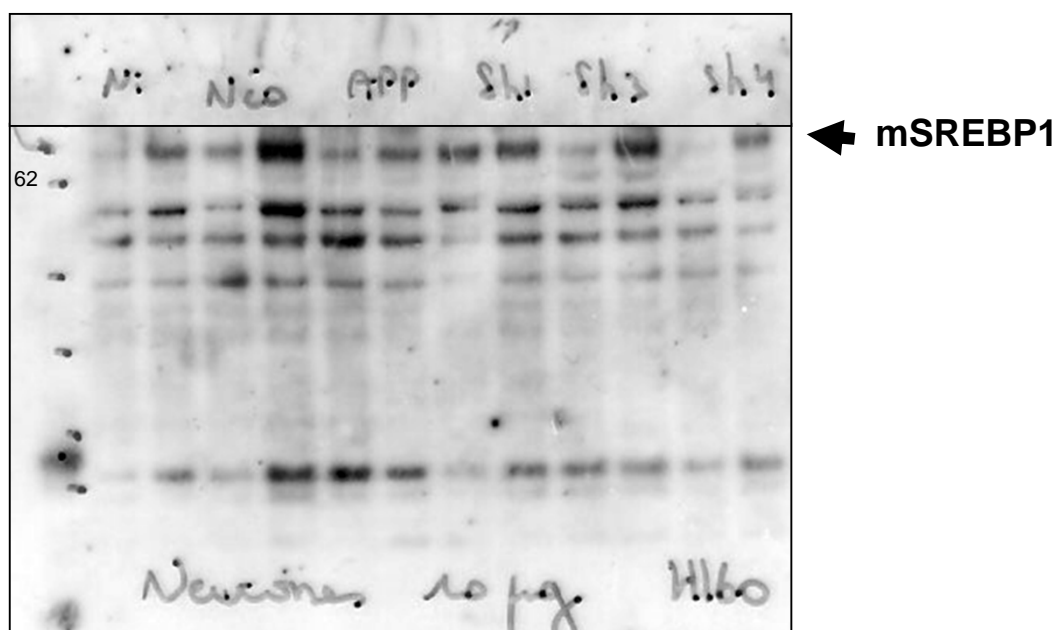

Fig. 2M

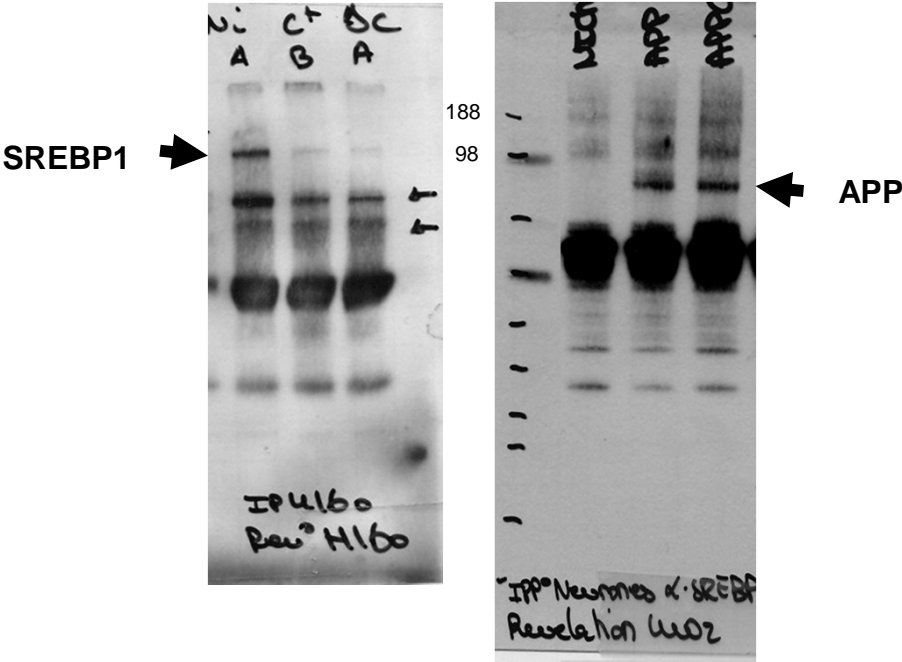

Fig. 20

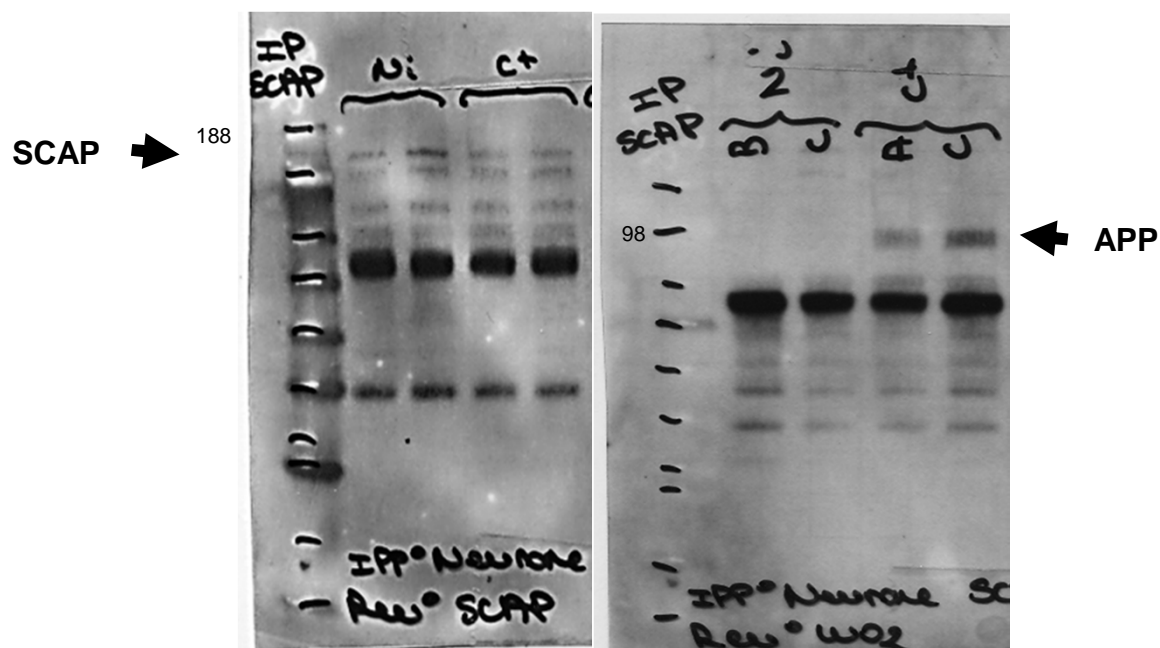

Fig. 2P

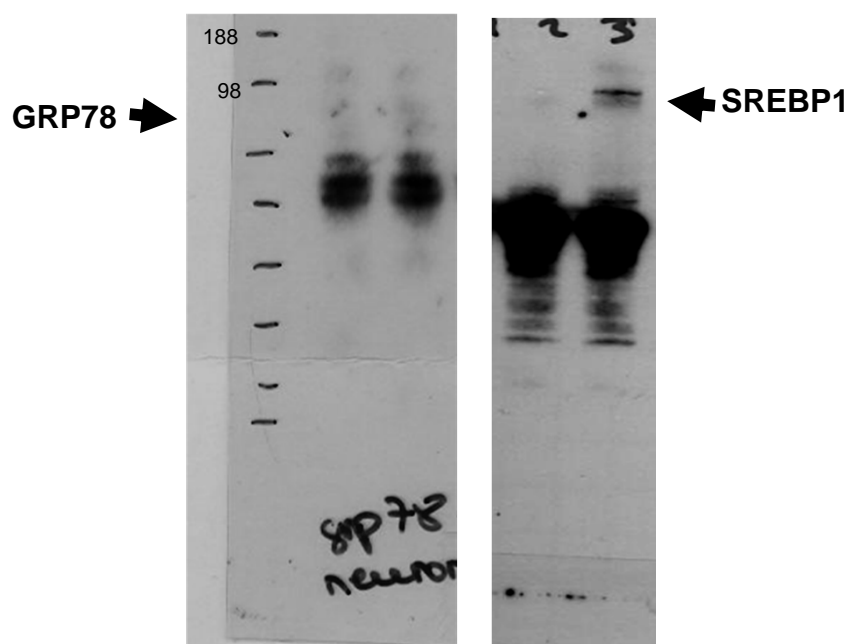

Supplement: Supplementary file 3 [file emmm0005-0608-sd3.pdf]

Fig. 3A

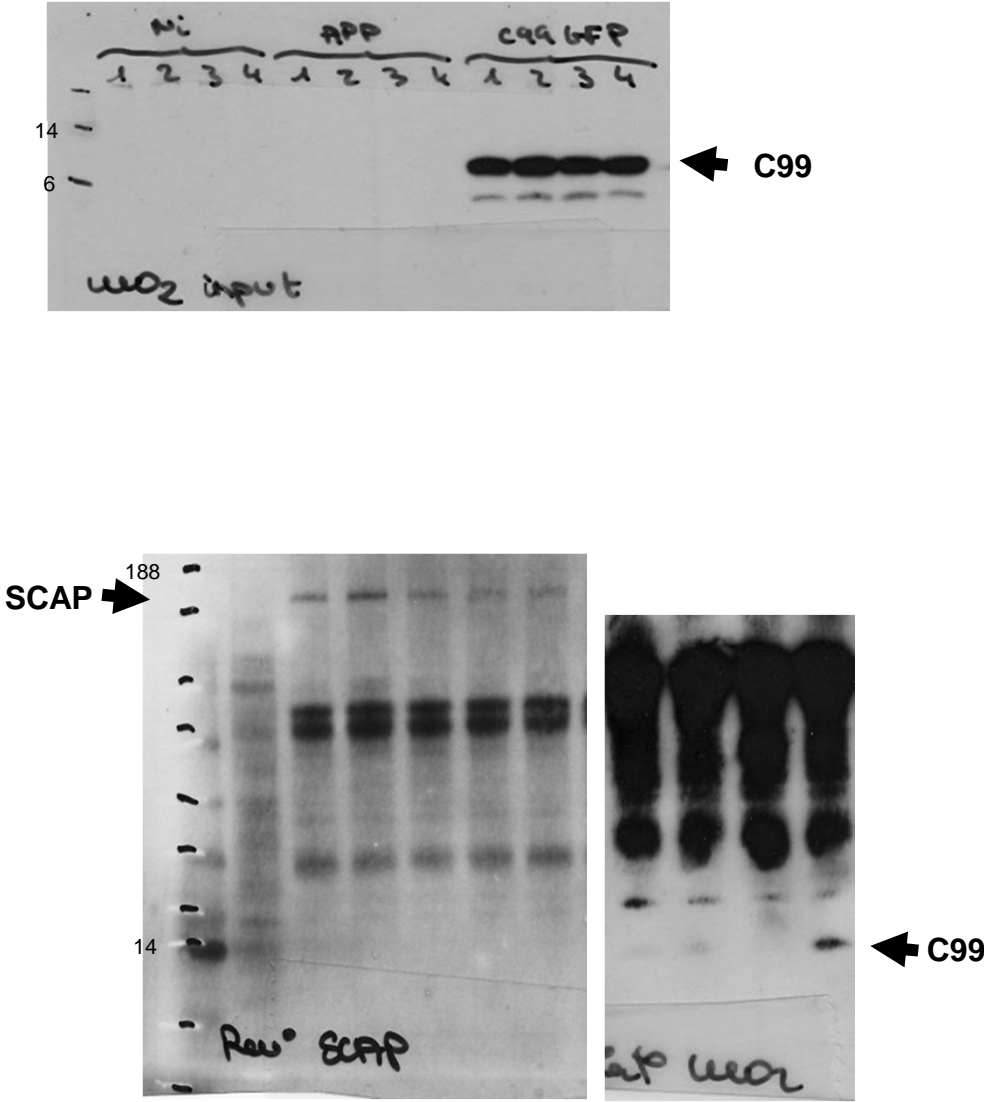

Fig. 3C

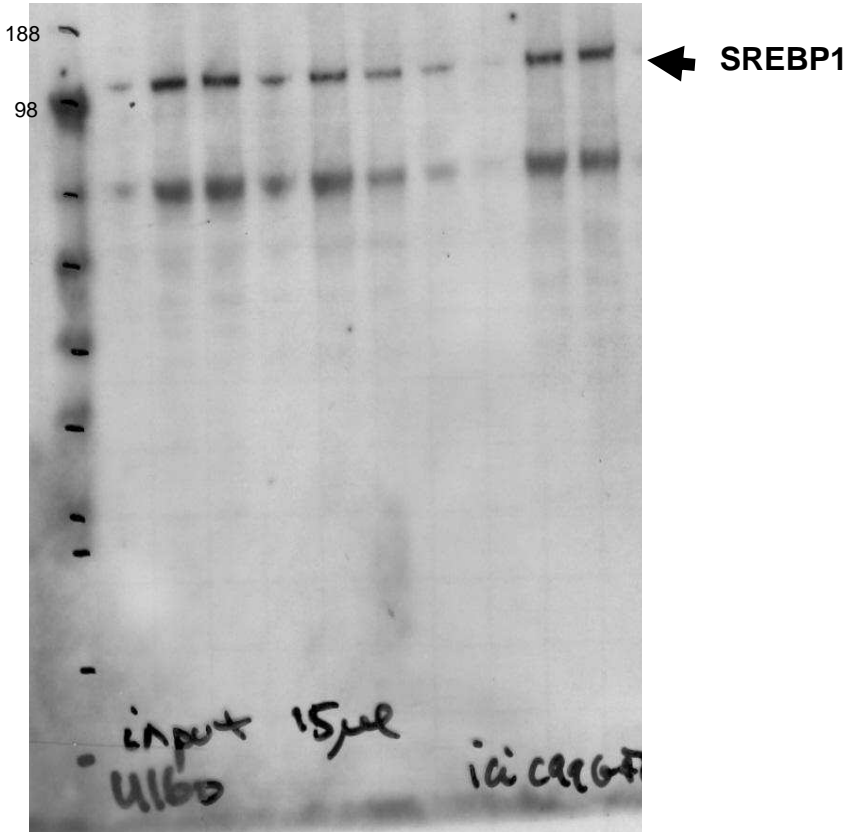

Fig. 3F

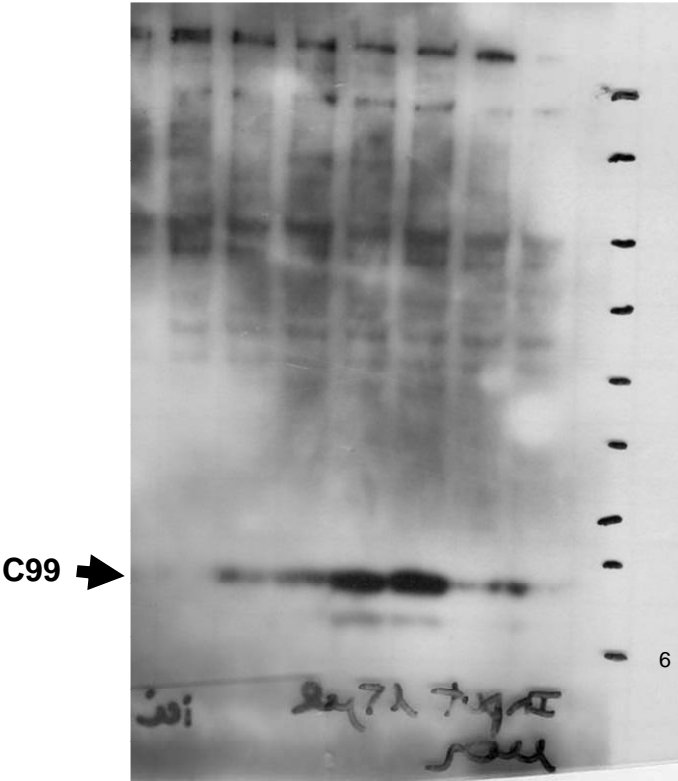

Supplement: Supplementary file 4 [file emmm0005-0608-sd4.pdf]

Fig. 4B

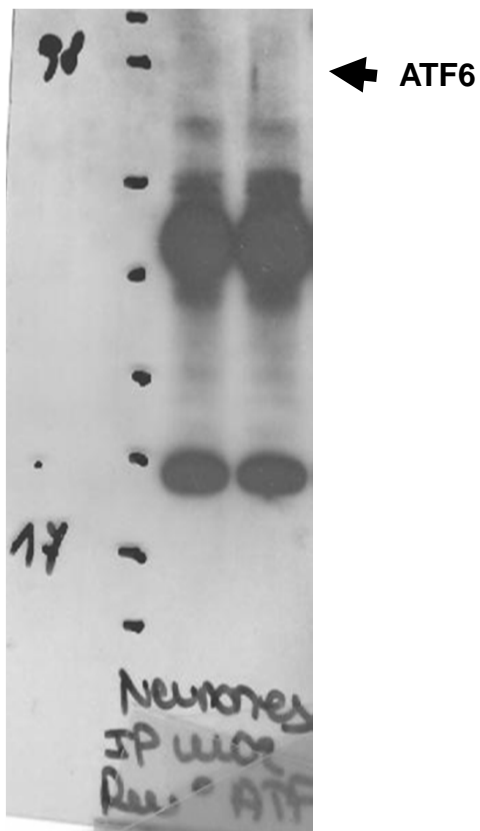

Fig. 4C

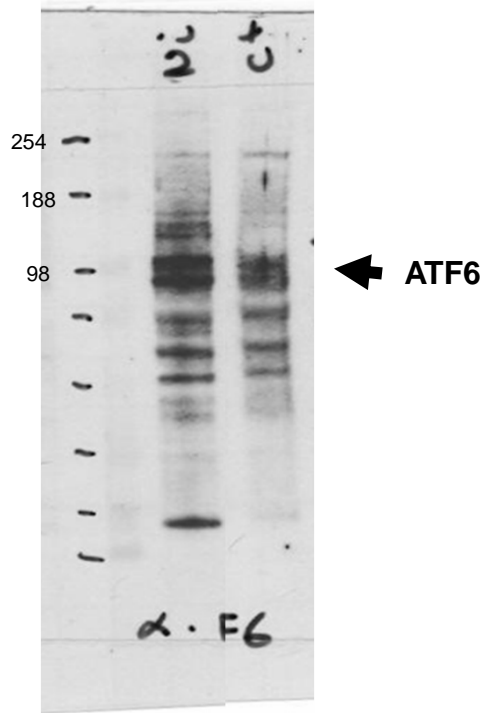

Supplement: Supplementary file 5 [file emmm0005-0608-sd5.pdf]

Fig. 5D and E

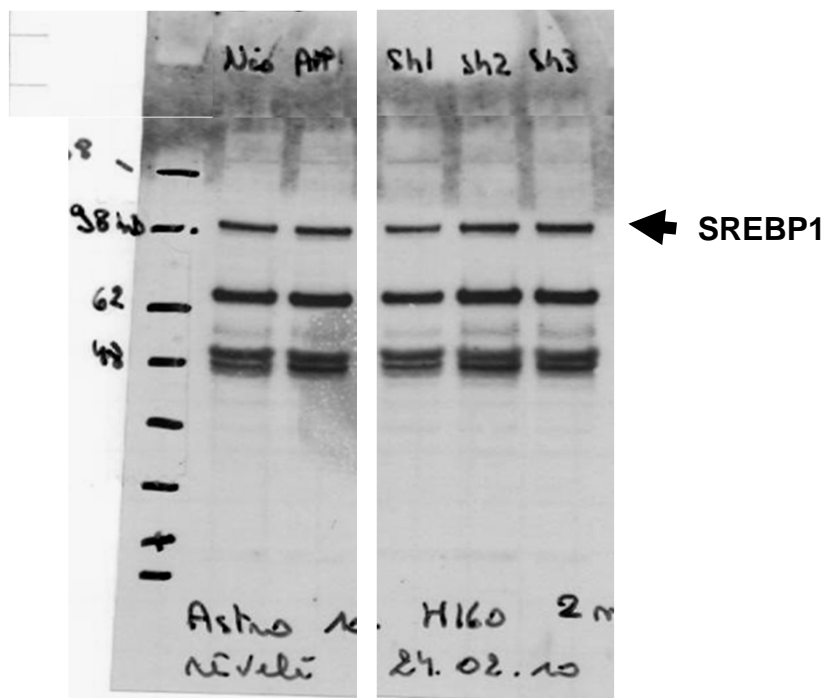

Fig. 5E

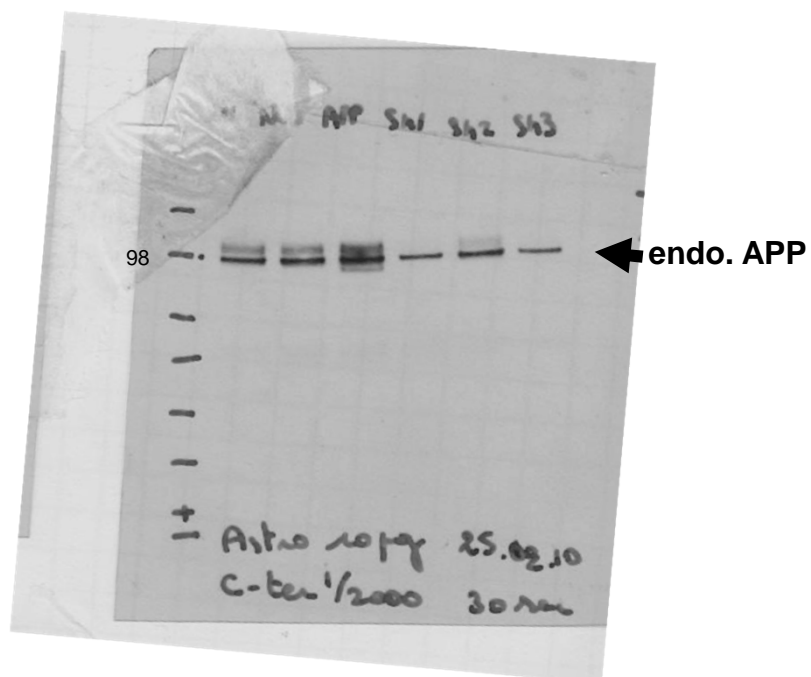

Fig. 5F

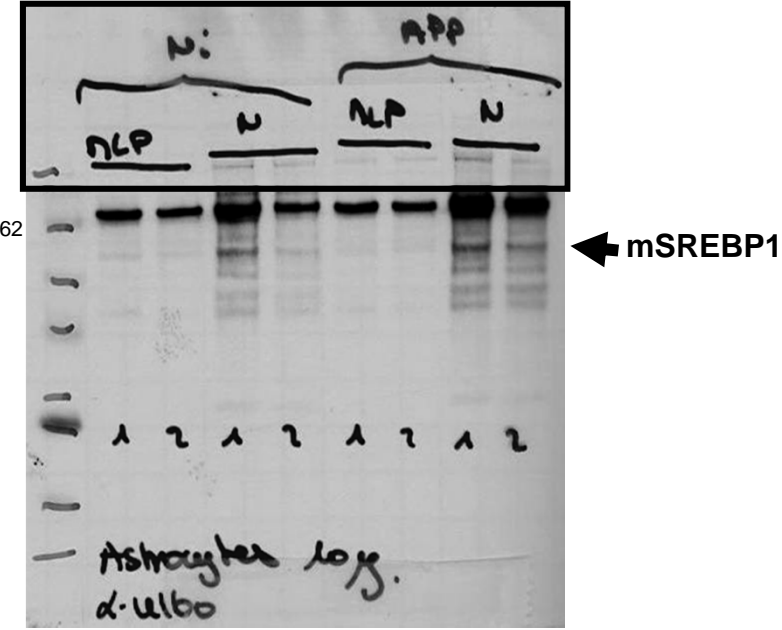

Fig. 5S

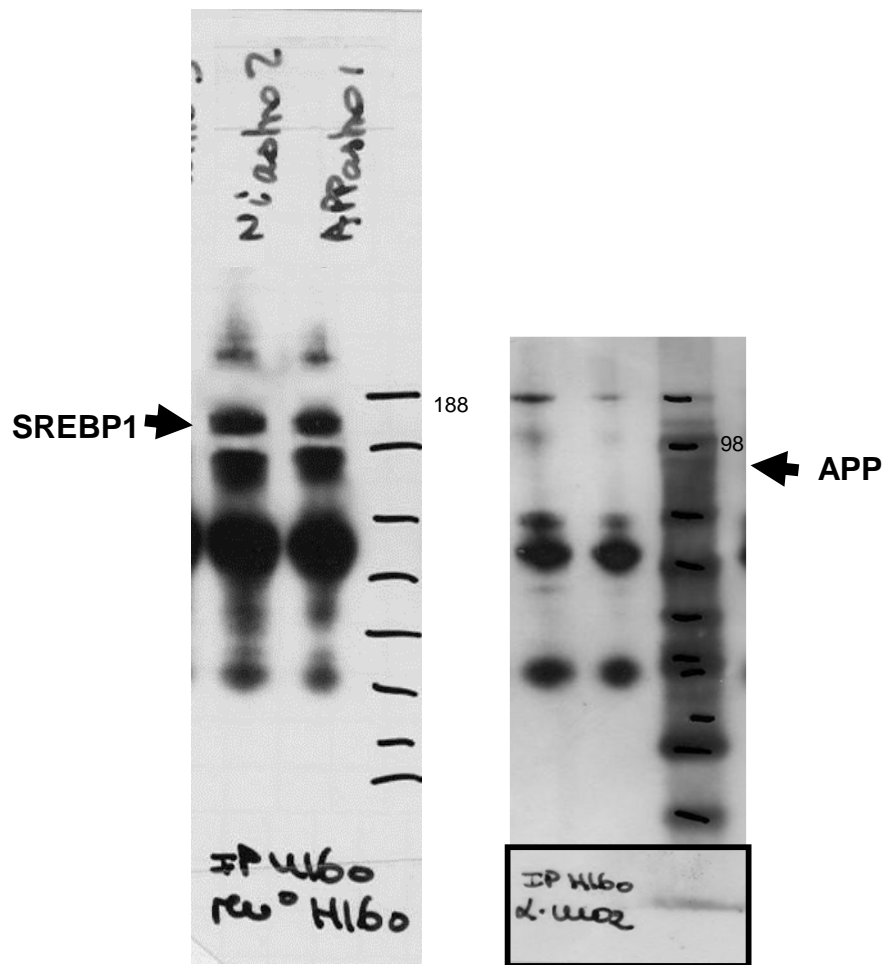

Supplement: Supplementary file 6 [file emmm0005-0608-sd6.pdf]

Fig. 6A

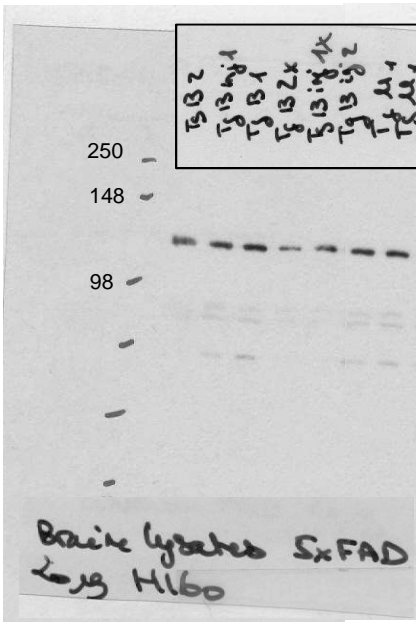

← SREBP1

Tris-Glycine gel 8%  
15 pishes 20 μg

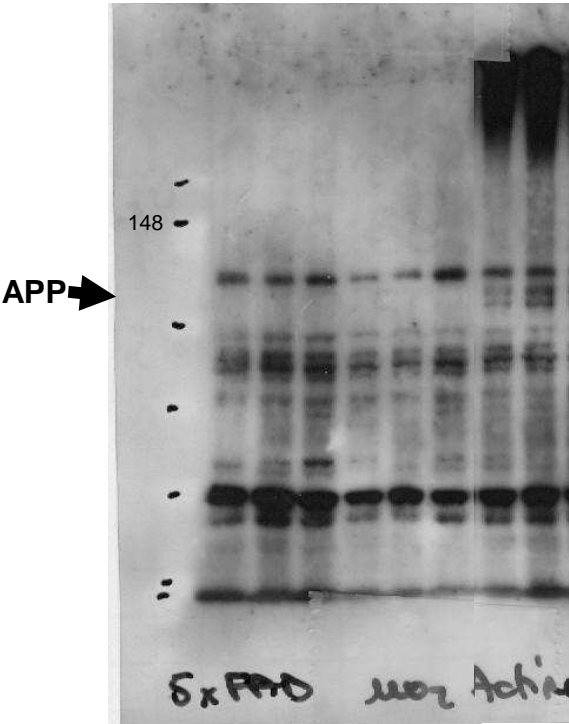

APP →

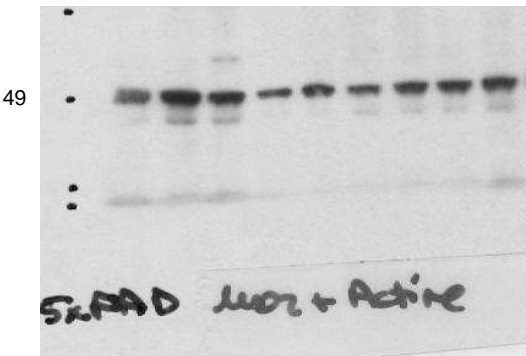

← actin

Fig. 6C

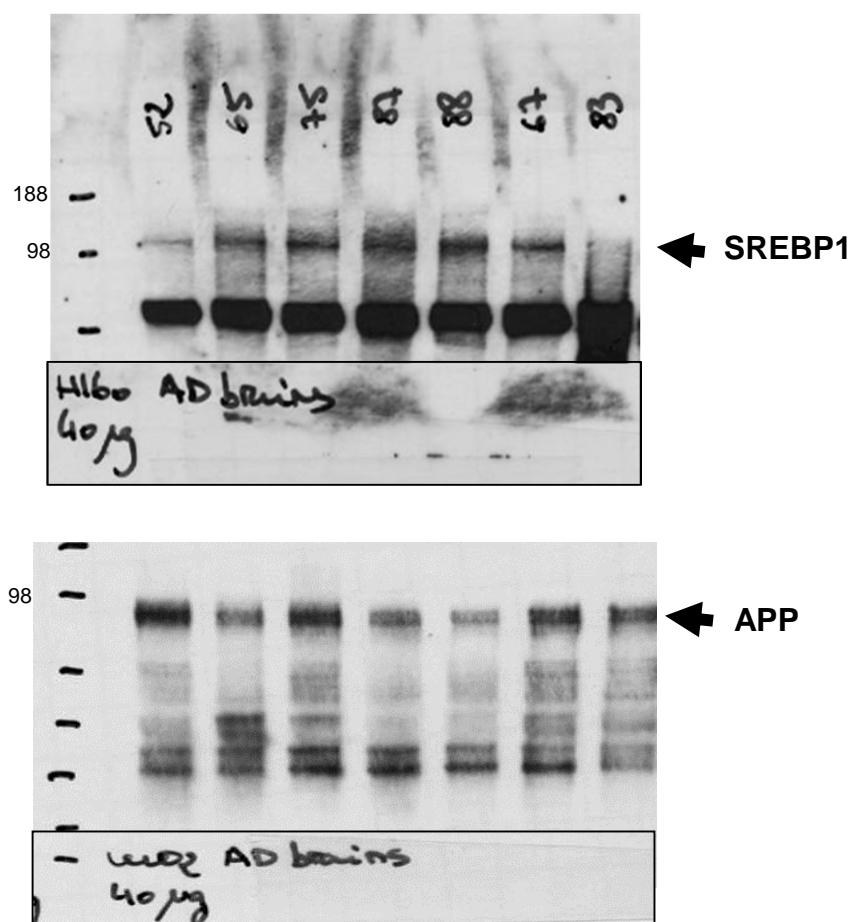

**Fig. 6D**

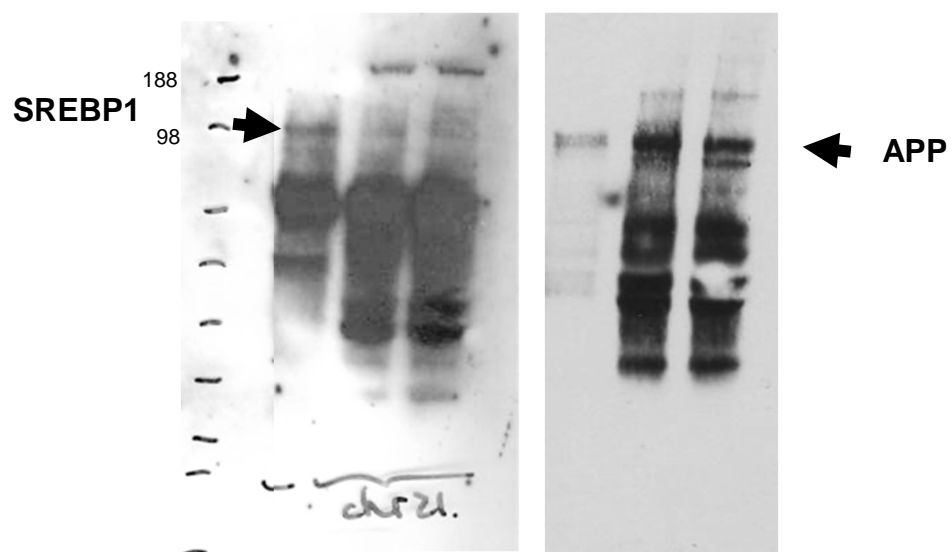

Supplement: Supplementary file 7 [file emmm0005-0608-sd7.pdf]

Fig. 7G

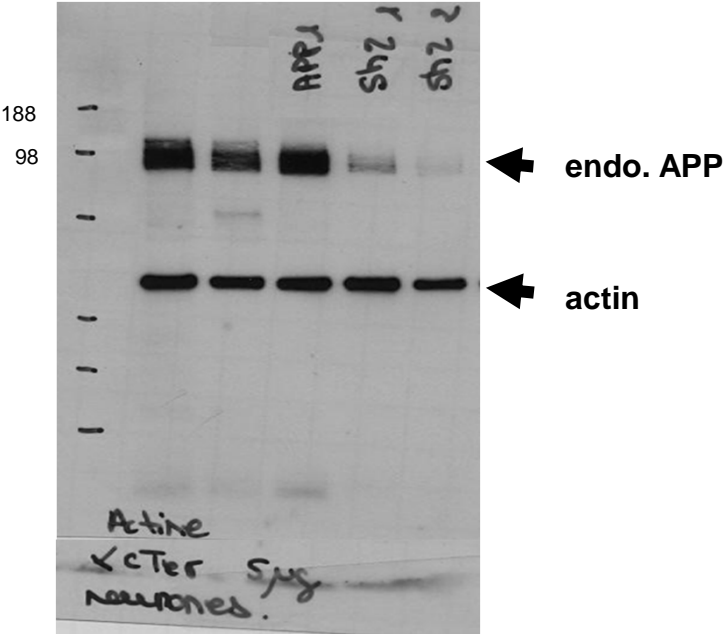

Supplement: Supplementary file 8 [file emmm0005-0608-sd8.pdf]
