# Supplementary material for: Amyloid precursor protein controls cholesterol turnover needed for neuronal activity
Source: EMBO Mol Med. 2013 Apr 2;5(4):608–25. doi: 10.1002/emmm.201202215 (PMC3628100; doi:10.1002/emmm.201202215)
Supplement: Supplementary file 9 [file emmm0005-0608-sd9.pdf]

Supporting information

Table of Content:

- Supplementary figures and legends pages 2-8:
  - Supporting information Fig.1 page 2
  - Supporting information Fig.2 page 3
  - Supporting information Fig.3 page 4
  - Supporting information Fig.4 page 5
  - Supporting information Fig.5 page 6
  - Supporting information Fig.6 page 7
  - Supporting information Fig.7 page 8
- Supplementary methods pages 9
- Supplementary references page 10

Supplementary figures

Supporting information Fig.1

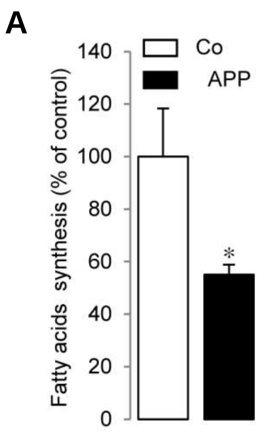

Supporting information Fig. 1. APP expression decreases fatty acids synthesis.

A. Fatty acids synthesis was measured by ^{14}C acetate incorporation in neurons expressing APP compared to Co (n=4). Unpaired Student's *t* statistical test (*P* = 0.03).

Supporting information Fig.2

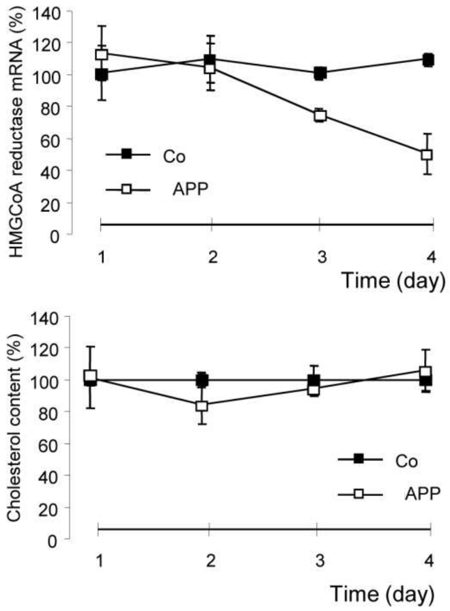

Supporting information Fig. 2. Decrease of HMG-CoA gene transcription does not result from a transient cholesterol overload in APP expressing neurons.

Primary rat cortical neurons were infected or not (control, Co) by AdhAPP (APP). HMG-CoA reductase mRNA levels in Co and APP neurons were analysed by qRT-PCR (n=6) from day 1 to day 4 of APP expression (upper panel). Results (mean ± SE) are normalized by GAPDH mRNA and expressed as percentage of Co. Cholesterol content (lower panel) in cell membranes (n=6) was measured in Co and APP expressing neurons. Results (mean ± SE) are given as percentage of Co neurons.

Supporting information Fig. 3

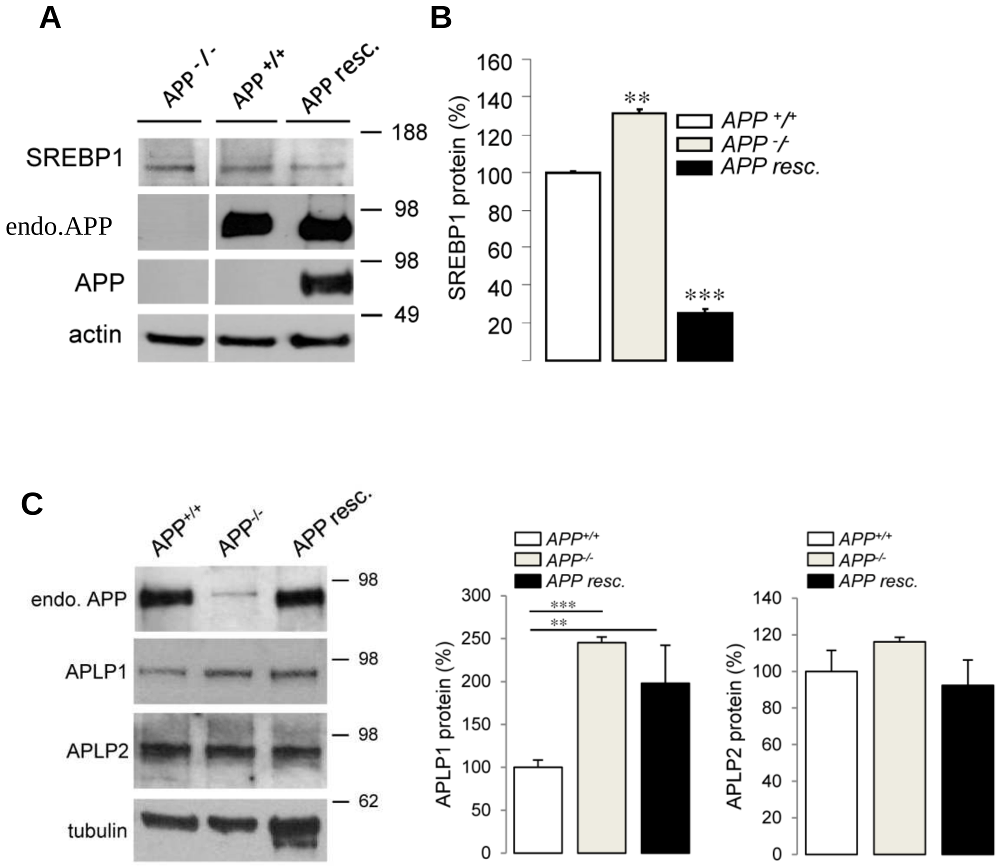

Supporting information Fig. 3. Knockout of endogenous APP leads to the accumulation of SREBP1.

A. Western blots of cell lysates from primary cultures of mouse cortical neurons prepared from wild type (*APP*$^{+/+}$), *APP* knockout mice *(APP*$^{-/-}$*)*, and *APP*$^{-/-}$ neurons infected by AdhAPP (*APP resc*.).

B. Quantification of SREBP1/actin ratios; results were expressed as percentage of *APP*$^{+/+}$ (n=3) (∗∗*P*< 0.01; ∗∗∗*P*< 0.001).

C. Western blots of cell lysates from primary cultures of mouse cortical neurons prepared from wild type (*APP*$^{+/+}$), *APP* knockout mice *(APP*$^{-/-}$*)*, and *APP*$^{-/-}$ neurons infected by AdhAPP (*APP resc*.) (left panel); quantification of APLP1 and APLP2/tubulin ratios; results were expressed as percentage of *APP*$^{+/+}$ (n=3) (∗∗*P*< 0.01; ∗∗∗*P*< 0.001) (right panels).

Supporting information Fig. 4

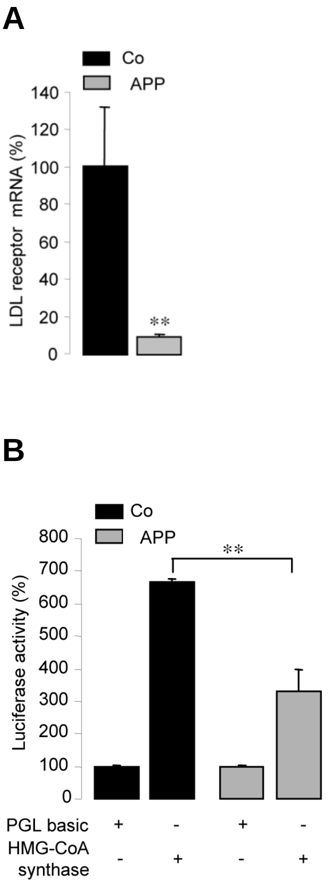

Supporting information Fig. 4. APP expression down-regulates *LDL receptor* and *HMG-CoA synthase* transcription.

A. Comparative qRT-PCR analysis of LDL receptor mRNA in control (Co) and APP expressing neurons. Results (mean ± s.e.m, n=6) are normalized by GAPDH mRNA and expressed as percentage of Co.

B. The transactivation of the *HMG-CoA synthase* gene promoter fused to a luciferase reporter gene was analysed in a luciferase reporter assay. Control (Co) and APP expressing neurons were transfected with the pGL3 (PGL basic) or pGL2-HMG-CoA synthase luciferase plasmids, and the luciferase activity was measured 48h after transfection. Results (mean ± s.e.m, n=3) are expressed as percentage of PGL basic luminescence (***P*< 0.01).

Supporting information Fig.5

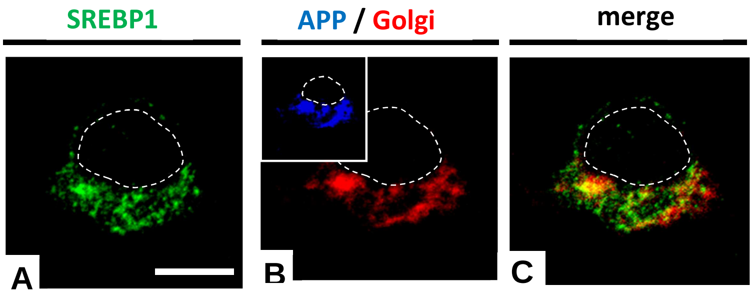

Supporting information Fig. 5. Full length SREBP1 co-localizes with APP in the Golgi.

A-C. Comparison of the cellular localization of SREBP1 (anti-C-terminus antibody, green), TGN46 (red) and APP (WO2 antibody, blue) in APP-expressing neurons.

C. Merged image. Scale bar: 5µm.

Supporting information Fig. 6

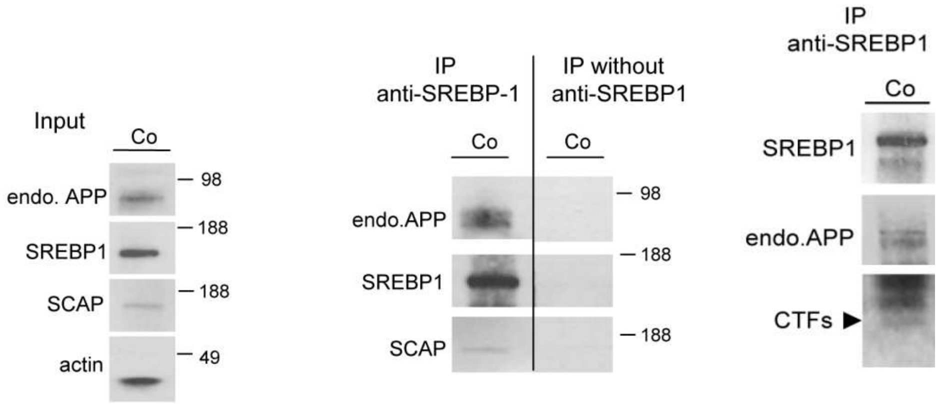

Supporting information Fig. 6. Endogenous APP and APP CTFs interact with SREBP1. Cell lysates from control neurons (Co) were analysed by Western blotting with anti-N-terminus SREBP1, anti-APP C-terminal, for the detection of endogenous APP (endo. APP) and APP carboxy-terminal fragments (CTFs), anti-SCAP, a cargo protein of SREBP1 and anti-actin antibodies (input), or further immunoprecipitated (IP) with or without the anti-Nterminus SREBP1 antibody and analyzed in Western blotting using anti-APPCterminal, -SCAP or -SREBP1 antibodies.

Supporting information Fig. 7

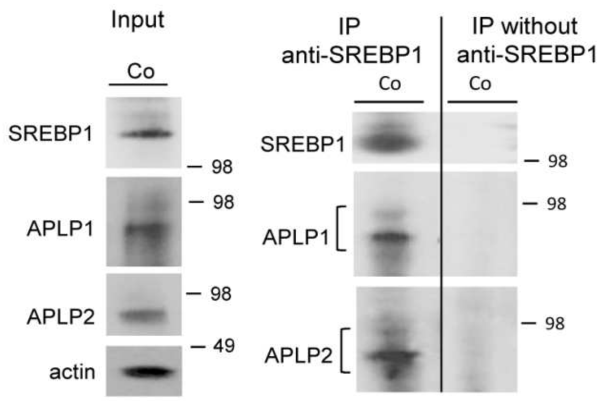

Supporting information Fig. 7. Endogenous APLP1 and APLP2 interact with SREBP1. Cell lysates from control neurons (Co) were analysed by Western blotting with anti-N-terminus SREBP1, anti-APLP1, anti-APLP2 and anti-actin antibodies (input), or further immunoprecipitated (IP) with or without the anti-Nterminus SREBP1 antibody and analyzed in Western blotting.

Supplementary methods

Fatty acids synthesis was measured four days after infection and was performed as described for cholesterol synthesis in the materials and methods section of the paper.

Cholesterol extraction and assay were performed as described in the materials and methods section of the paper.

RNA extraction and real time PCR were performed as described in the Methods section of the paper. The primers used were the following (**F**, Forward primer; **R**, Reverse primer):
LDL receptor **F**-5’AAGACCACGGAGGACGAGATC3’,
R-5’CCTCCAGGCTGACCATCTGT3’;
GAPDH **F-**5’CCCCCAATGTATCCGTTGTG3’, **R-**5’TGATTTCCCGTAGGACCGAT3’;

Transient transfection and luciferase activity

Four days after infection, neuronal cultures (4×10^5 cells/cm^2) were transfected using Lipofectamine 2000 (Invitrogen) with pGL3-Basic luciferase or pGL2-HMG-CoA synthase luciferase plasmids (Demoulin *et al.*, 2004) along with the control *Renilla* luciferase reporter vector (phRG-TK, Promega, Madison, WI) to correct for variations in transfection efficiency. Neurons were transfected with 1 μg of pGL3-Basic or pGL2-HMG-CoA synthase luciferase vectors and 0.17 μg of phRG-TK. Luciferase activity was measured 48 h after transfection with the dual-luciferase assay system (Promega). Luciferase activities are expressed in relative values and represented as percentage of control corresponding to pGL3-Basic alone.

Immunocytochemical analysis was performed as described in the experimental procedures section of the paper. Neurons were incubated in the same solution for 1 h at room temperature in the presence of the C20 (1: 100) antibody, directed against the C-terminus of SREBP1 with the WO-2 (1:1.000) antibody together with the sheep polyclonal anti-TGN46 (1: 50) antibody.

Protein analysis

Cell lysates (10 μg proteins) were analyzed by Western blotting as described in the experimental procedures section of the paper.

REFERENCE

1. Demoulin JB, Ericsson J, Kallin A, Rorsman C, Ronnstrand L, and Heldin CH (2004) Platelet-derived growth factor stimulates membrane lipid synthesis through activation of phosphatidylinositol 3-kinase and sterol regulatory element-binding proteins. J Biol Chem, 279, 35392-35402.
